# Supplementary material for: Multiomic analysis of human kidney disease identifies a tractable inflammatory and pro-fibrotic tubular cell phenotype
Source: Nat Commun. 2025 May 22;16:4745. doi: 10.1038/s41467-025-59997-4 (PMC12095627; doi:10.1038/s41467-025-59997-4)
Supplement: Supplementary file 1 — Supplementary Information [file 41467_2025_59997_MOESM1_ESM.pdf]

a

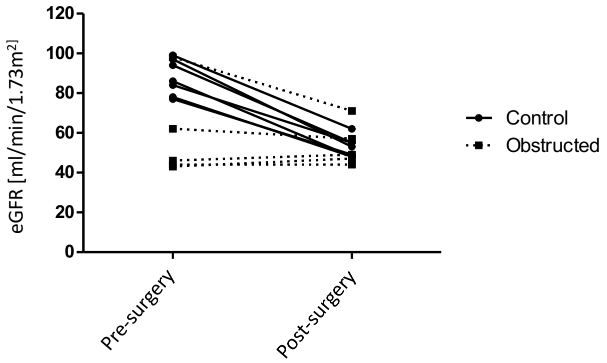

b

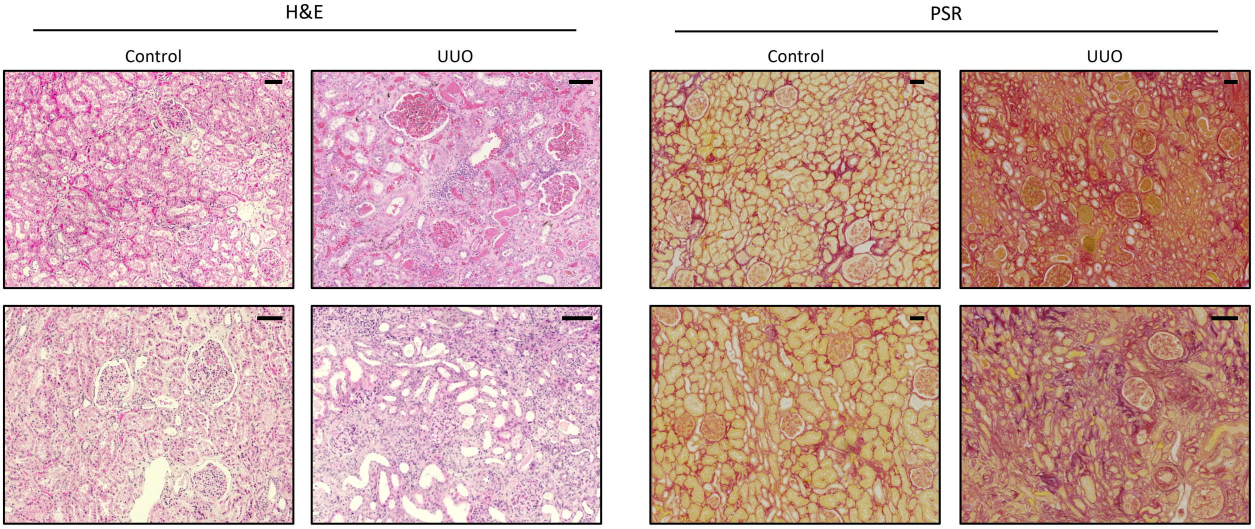

Fig. S1. Representative histology and renal function of controls and obstructed kidneys.

a) Estimated glomerular filtration rate (eGFR) readings pre- and post-nephrectomy in patients with unobstructed (*n* = 7) and obstructed kidneys (*n* = 5). Four of five obstructed samples show no change in eGFR, indicating they were not contributing to renal function.

b) Representative picrosirius red (PSR) and haematoxylin and eosin (H&E) images of two control and two UUO human nephrectomy samples. Left Panel: H&E stain. Right panel: PSR stain. Scale bars, 100 µm.

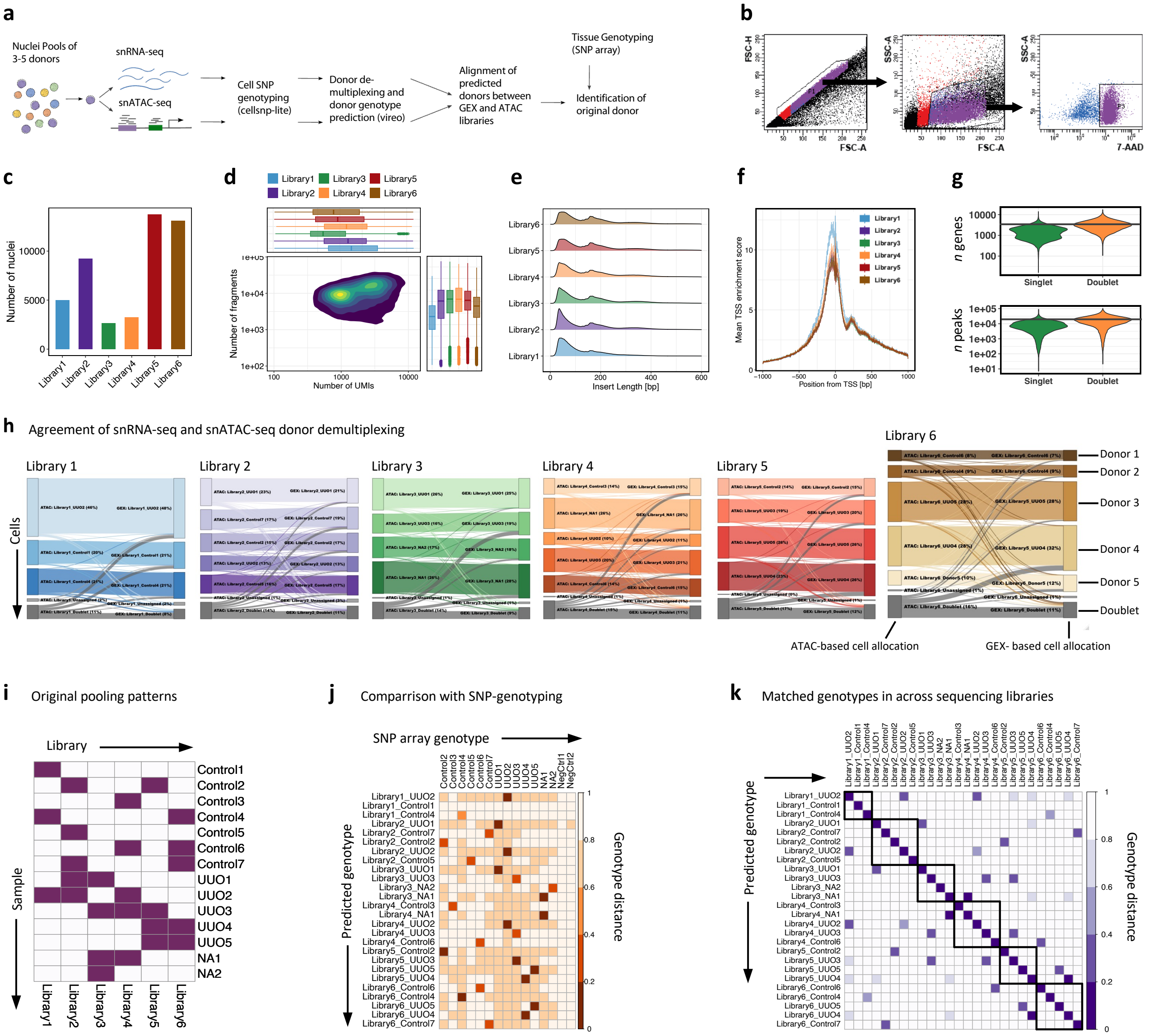

Fig. S2. Library quality metrics and donor multiplexing.

a) Schematic representation of the SNP-based sample pooling and demultiplexing strategy. Nuclei from different samples are pooled and GEX and ATAC libraries generated. SNPs present within sequencing reads are used to computationally allocate cells to predicted donors. Donors are initially predicted independently in ATAC and GEX libraries and later aligned to resolve conflicting donor predictions. SNP array genotyping is used to confirm the identity of samples.

b) Representative images of flow cytometric sorting strategy used to isolate nuclei. The nuclei suspension was filtered using a singlet and size gate before gating on 7-AAD^{+} events.

c) Barplot showing the number of nuclei obtained per library, post-filtering.

d) Density graph showing the number of unique molecular identifiers (UMIs) and chromatin fragments of recovered cells. Box plots on the top and right indicate the distribution in each library.

e) Density graph showing the length of recovered fragments. The large proportion of reads < 100bp represent nucleosome-free regions, while larger fragments are indicative of nucleosome occupancy.

f) Transcription start site (TSS) enrichment plot of the aggregate distribution of reads centred on TSS demonstrating strong enrichment at the centre of the distribution indicating a high signal-to-noise ratio.

g) Violin plots of the number of genes or peaks (log-scales) per nucleus, grouped by nuclei predicted to be singlets or doublets (presence of SNPs allocated to distinct donors). Nuclei doublets have higher gene and peak content indicating correct identification.

h) Agreement of donor predictions based on ATAC (left nodes) and GEX (right nodes) reads for all libraries. Coordinates on the y-axis represent cell barcodes with lines joining the respective barcodes for the same patient in the other modality.

i) Heatmap of sample allocation to libraries during pooling. Coloured tiles indicate that the sample was present in the respective library.

j) Similarity of the ground-truth genotype obtained by SNP microarray of the original tissue (columns) and the predicted genotypes based on sequencing reads. The distance is calculated as the mean Hamming distance at shared locations and is subsequently scaled between zero and one.

k) Heatmap comparing the predicted genotypes of all donors. Cells from some donors were present in more than one pooled library (e.g. UUO2 was present in library 1, 2 and 4 (**Fig. S2i**) and there was a high level of concordance of genotypes between pools where the same donor is present. Distance is calculated as the mean Hamming distance at shared genomic locations. For visualisation, the mean distance of independent ATAC and GEX genotypes is used after scaling values between zero and one.

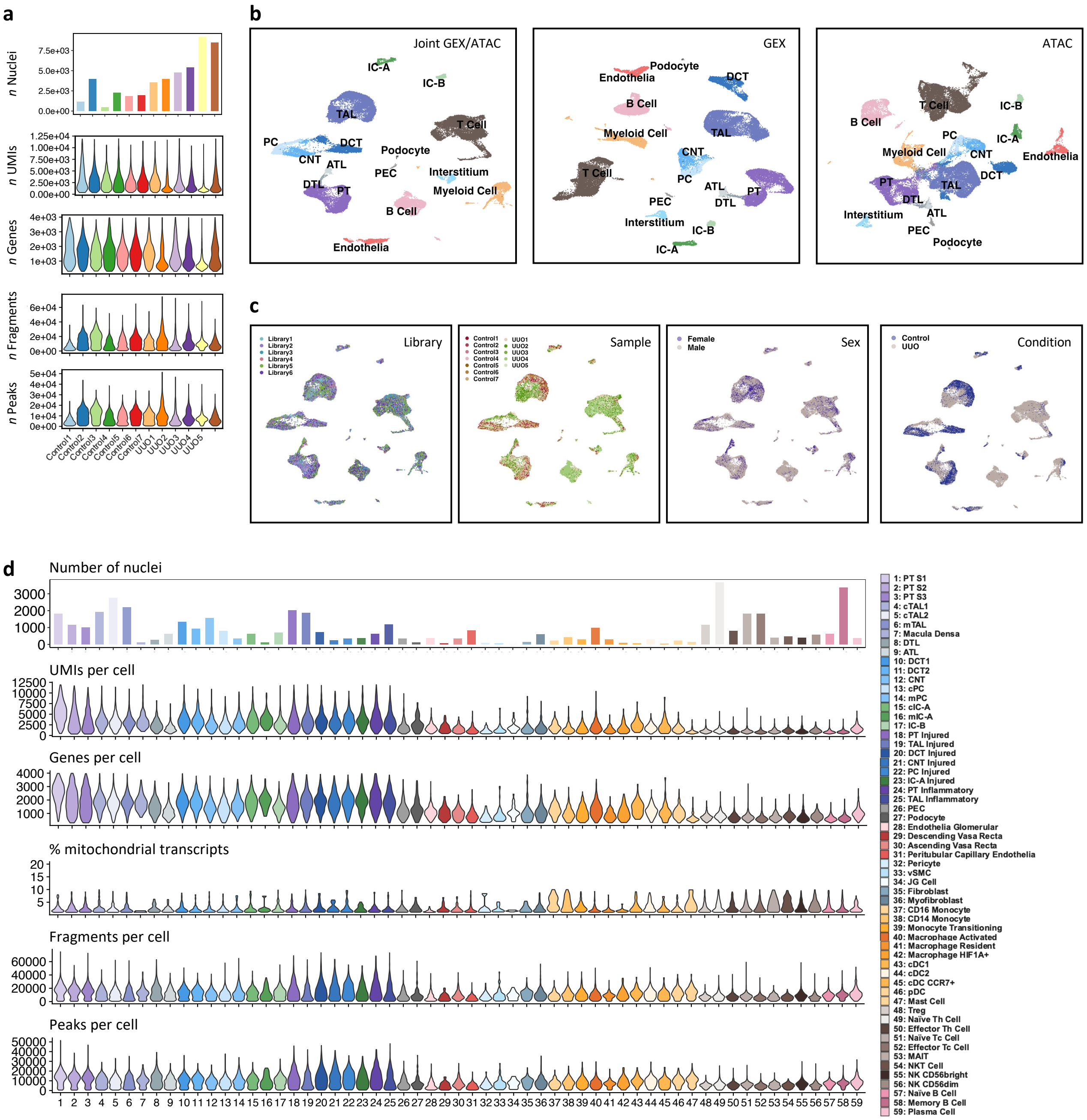

Fig. S3. Sample quality metrics and data integration.

a) Bar and violin plots showing the number of nuclei per sample passing quality metrics (number of detected UMIs, detected genes, chromatin fragments and number of peaks per cell).

b) UMAP based on the joint weighted nearest neighbour (WNN) embedding of GEX and ATAC modalities (**Fig. 1b**) (left) as well as independent GEX (middle) and ATAC (right) embeddings. Dimensionality reductions share common cell annotations, demonstrating agreement between GEX and ATAC modalities.

c) WNN UMAP (**Fig. 1b**) coloured by sequencing library, sample ID, sex of the donor and condition (control or obstructed).

d) Bar and violin plots showing the number of nuclei allocated to each cell type as well as associated quality metrics.

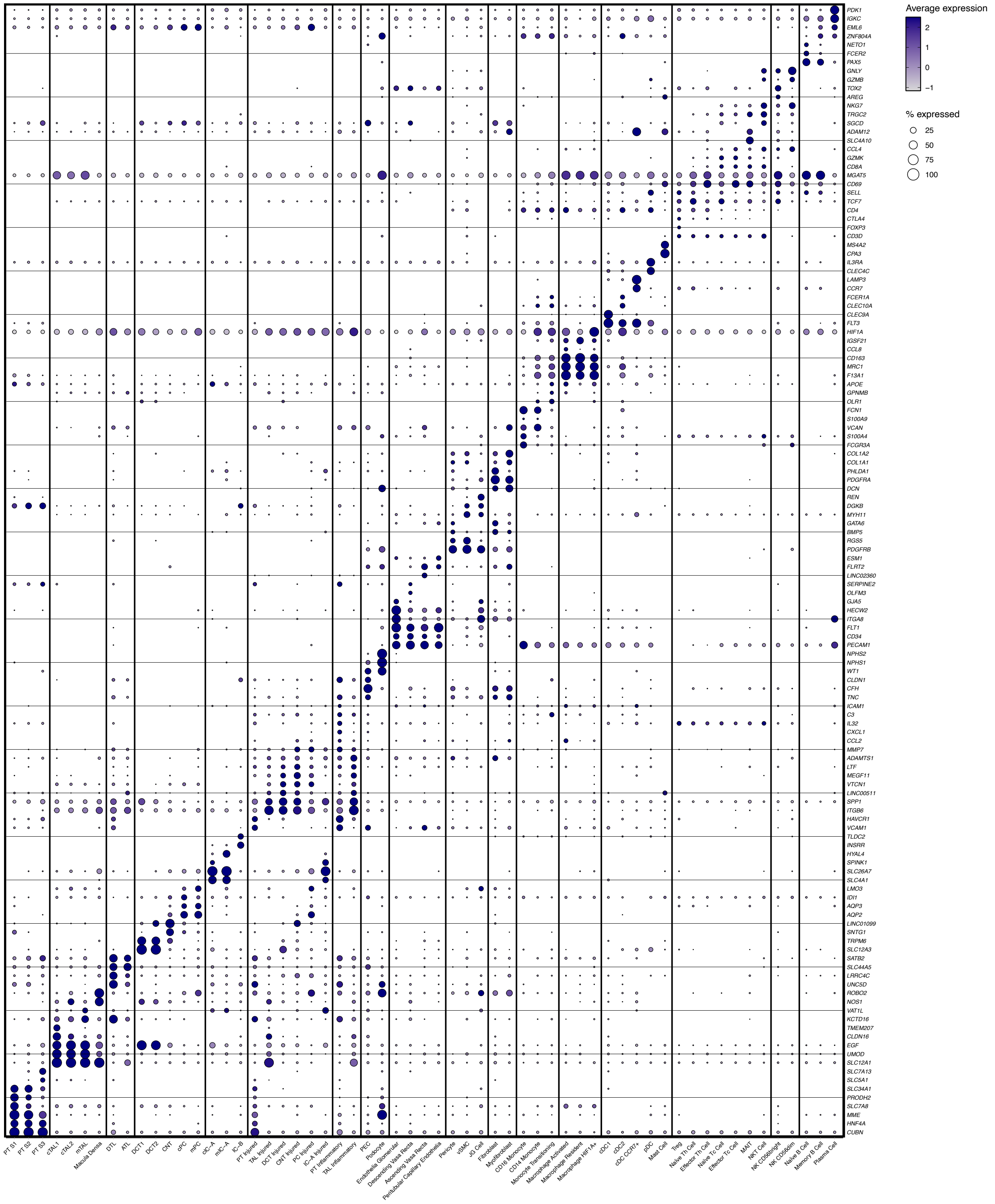

Fig. S4. snRNA-seq marker gene expression.

Dot plot showing expression of marker genes used to annotate clusters in **Fig. 1b**. Dot colours show the averaged gene expression values (log scale) and size indicates the proportion of cells expressing the gene.

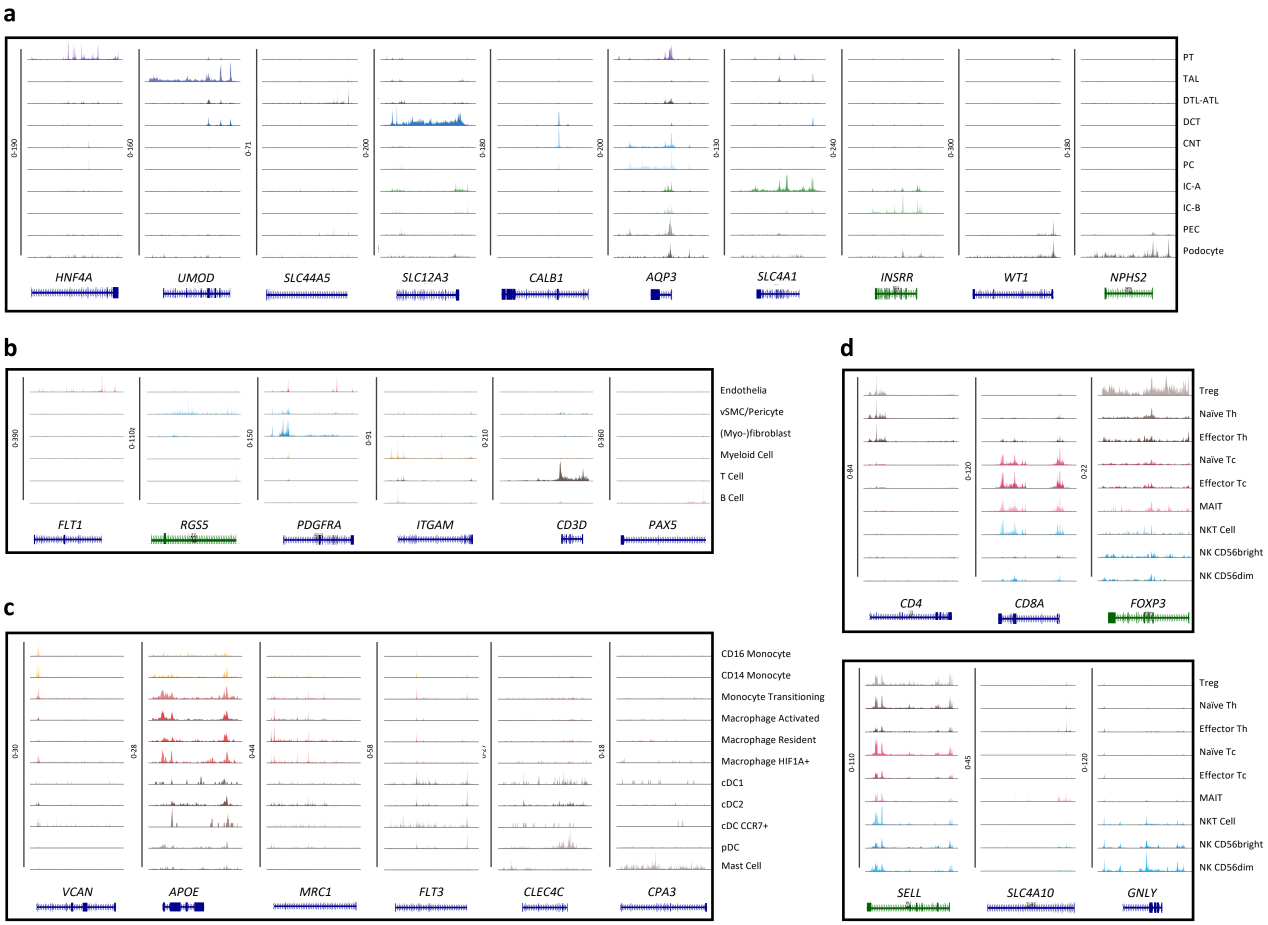

Fig. S5. snATAC-seq chromatin accessibility at marker gene loci.

Pseudo-bulk chromatin accessibility tracks associated with marker genes for **a)** Epithelial subsets; **b)** For interstitial, immune and endothelial subsets; **c)** For myeloid cell subsets; **d)** For T and NK cell subsets.

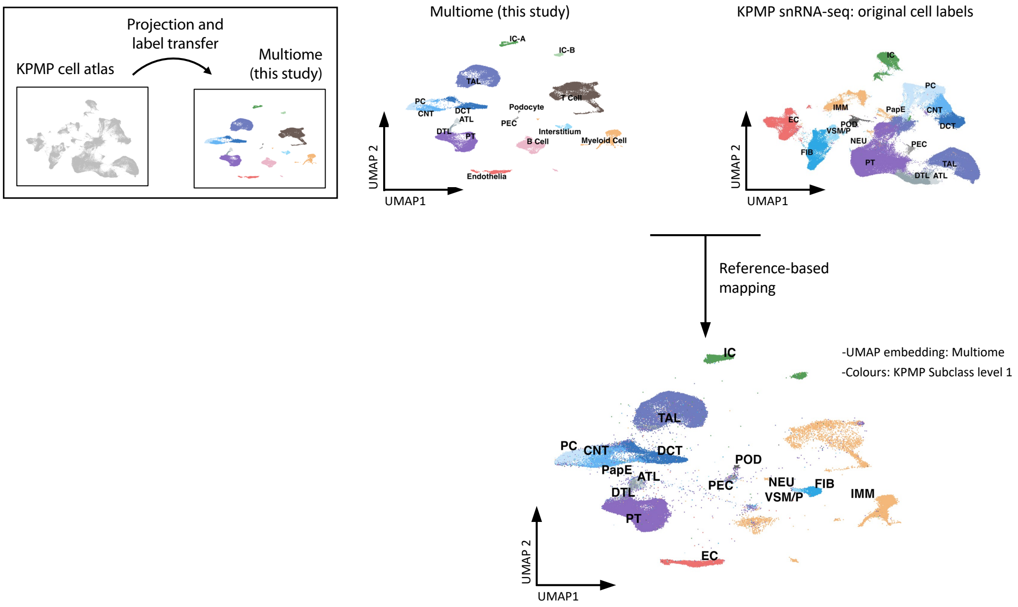

Fig. S6. Comparison of cell type annotations with Kidney Precision Medicine Project (KPMP) snRNA-seq atlas.
The KPMP sn-RNA dataset (172,847 nuclei) was projected onto the multiome WNN UMAP embedding to assign KPMP nuclei to the most closely aligning cluster in the multiome cell annotations. Cells are coloured by the original KPMP Subclass level 1 annotation (top right). The bottom plot shows KPMP transcriptomes projected onto the multiome UMAP, coloured according to original cell types showing broad agreement between annotations.
PEC, parietal epithelial cell; PT,proximal tubule; DTL, descending thin limb; ATL, ascending thin limb; TAL, thick ascending limb; DCT, distal convoluted tubule; CNT, connecting tubule; PC, principal cell; IC, intercalated cell; VSM/P vascular smooth muscle cell/pericyte; POD, podocyte; EC, endothelial cell; FIB, fibroblast; IMM, immune cell; PapE – papillary epithelial cell.

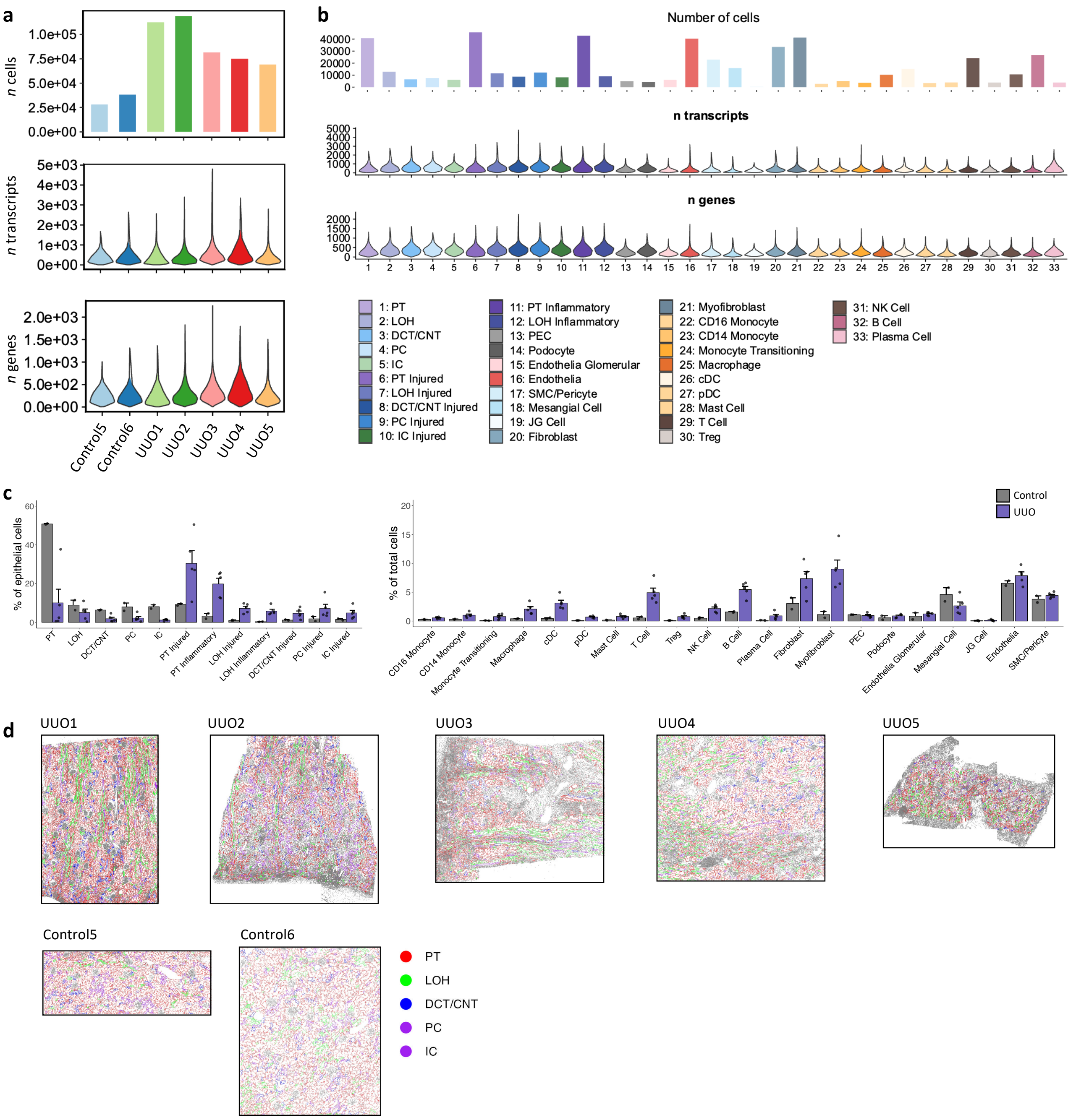

Fig. S7. CosMx (6,000-plex) quality metrics.

a) Bar and violin plots showing the number of cells per sample and number of detected transcripts and unique genes per cell.

b) Bar and violin plots showing the number of cells, genes and transcripts allocated to each fine cell type annotation.

c) Percentage of cells assigned to each cell type as a proportion of all epithelial cells (for epithelial clusters, left) and of total cells (for non-epithelial clusters, right) in obstructed (*n* = 5) and obstructed kidneys (*n* = 2). Plots show means ± SEM.

d) 2D spatial plots showing tissue section morphology as well as highlighting the tubular architecture. Individual points represent cells, coloured by tubular cell types. Grey dots are allocated to non-tubular cell types.

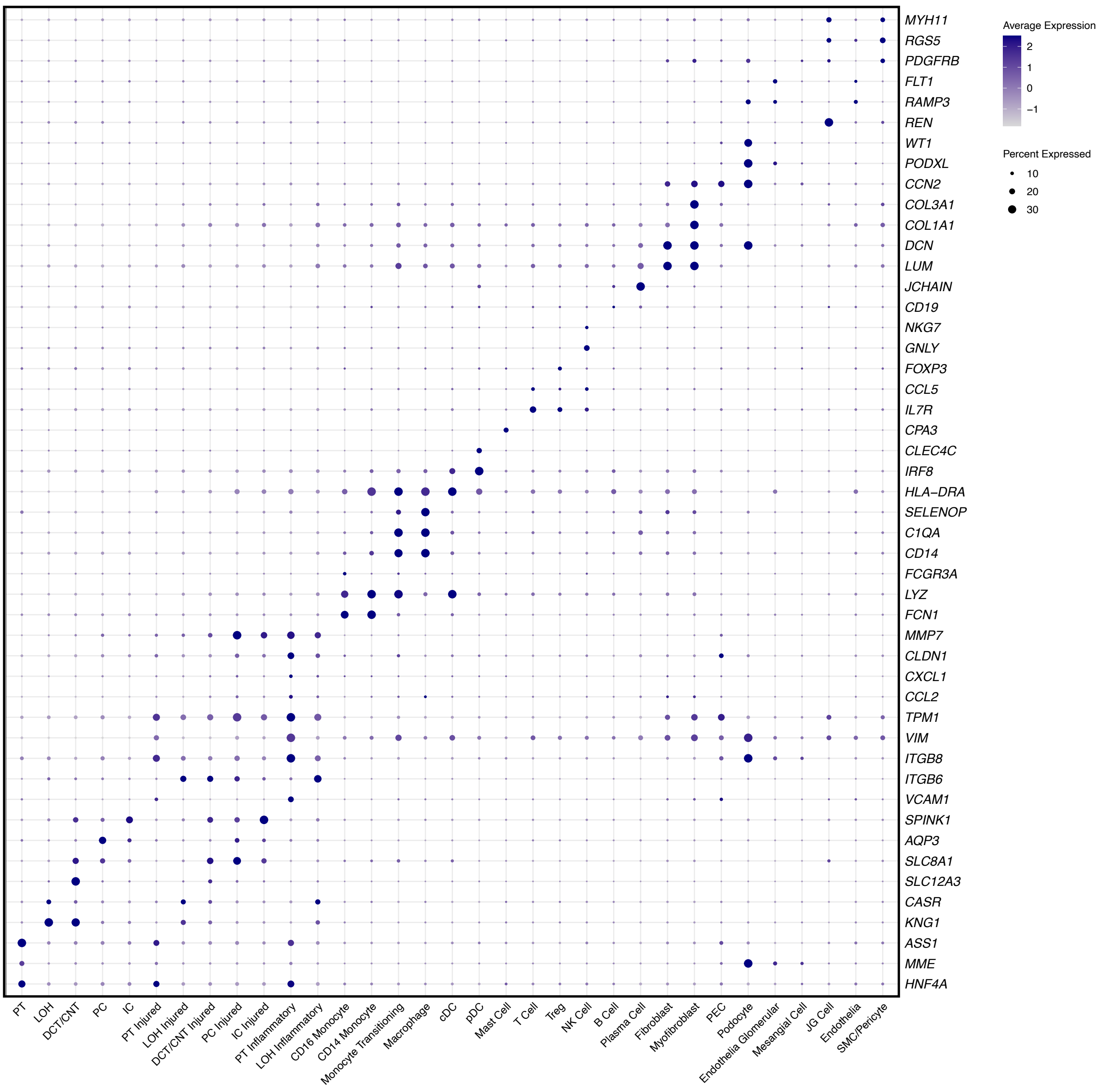

Fig. S8. CosMx (6,000-plex) cell type markers.

Dot plot showing expression patterns of differentially expressed genes of detailed cell clusters found in the CosMx dataset. Dot colours show the averaged gene expression values (log scale) and size indicates the proportion of cells expressing the gene.

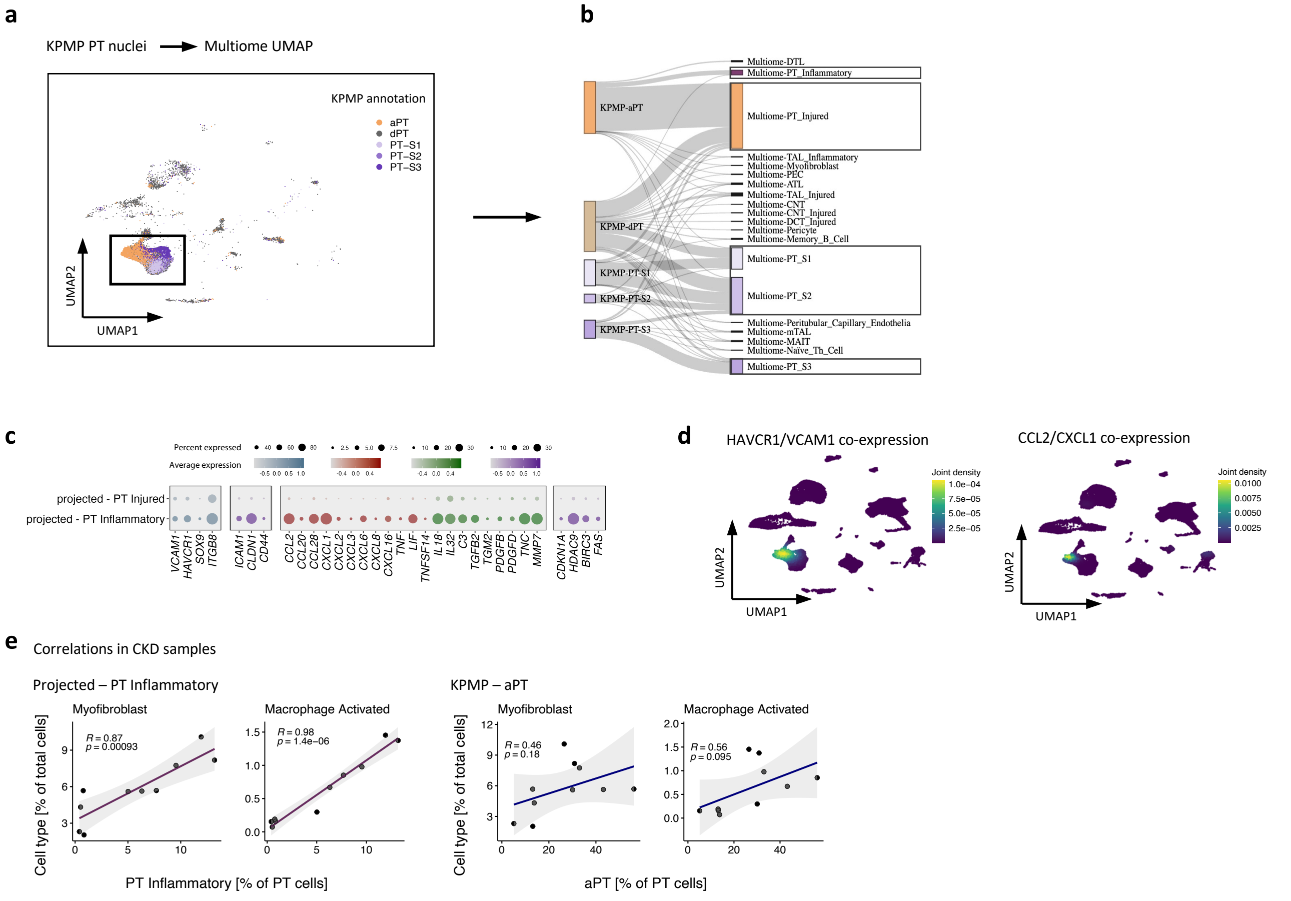

Fig. S9. Comparative analysis of PT cells in the KPMP snRNA-seq atlas (4)**.**

a) Subset of all KPMP PT cells (PT S1-S3, aPT, dPT) project onto the multiome UMAP (this study). The plot shows KPMP nuclei in projected locations on the multiome UMAP.

b) Sankey diagram showing the relationship of original KPMP subclass level-2 annotations and predicted multiome cell types for PT cells.

c) KPMP nuclei predicted as inflammatory PT show a similar gene expression signature to that observed in the equivalent cells in the multiome atlas. Transcriptomes are derived from KPMP nuclei and grouped according to projected annotations. Colours show the averaged gene expression values (log scale) and dot size indicates proportion expressed with scales indicated by the colour.

d) Co-expression of *HAVCR1* and *VCAM1* or *CCL2* and *CXCL1* in the multiome dataset. Colour indicates areas of higher co-expression.

e) Correlations of adaptive PT (aPT) or projected inflammatory PT abundance with key fibrosis mediators in the KPMP atlas. Samples are from patients with CKD (n = 10) and samples from patients with AKI and healthy controls have been excluded. Myofibroblast and activated macrophage proportions are derived from projected annotations and show proportions in relation to total cells in the sample. Inflammatory PT cells are derived from projected annotations, aPT cells refer to the original annotations. PT cell states are relative to total PT cell abundance. Graphs show linear regression plots and Pearson correlation co-efficient.

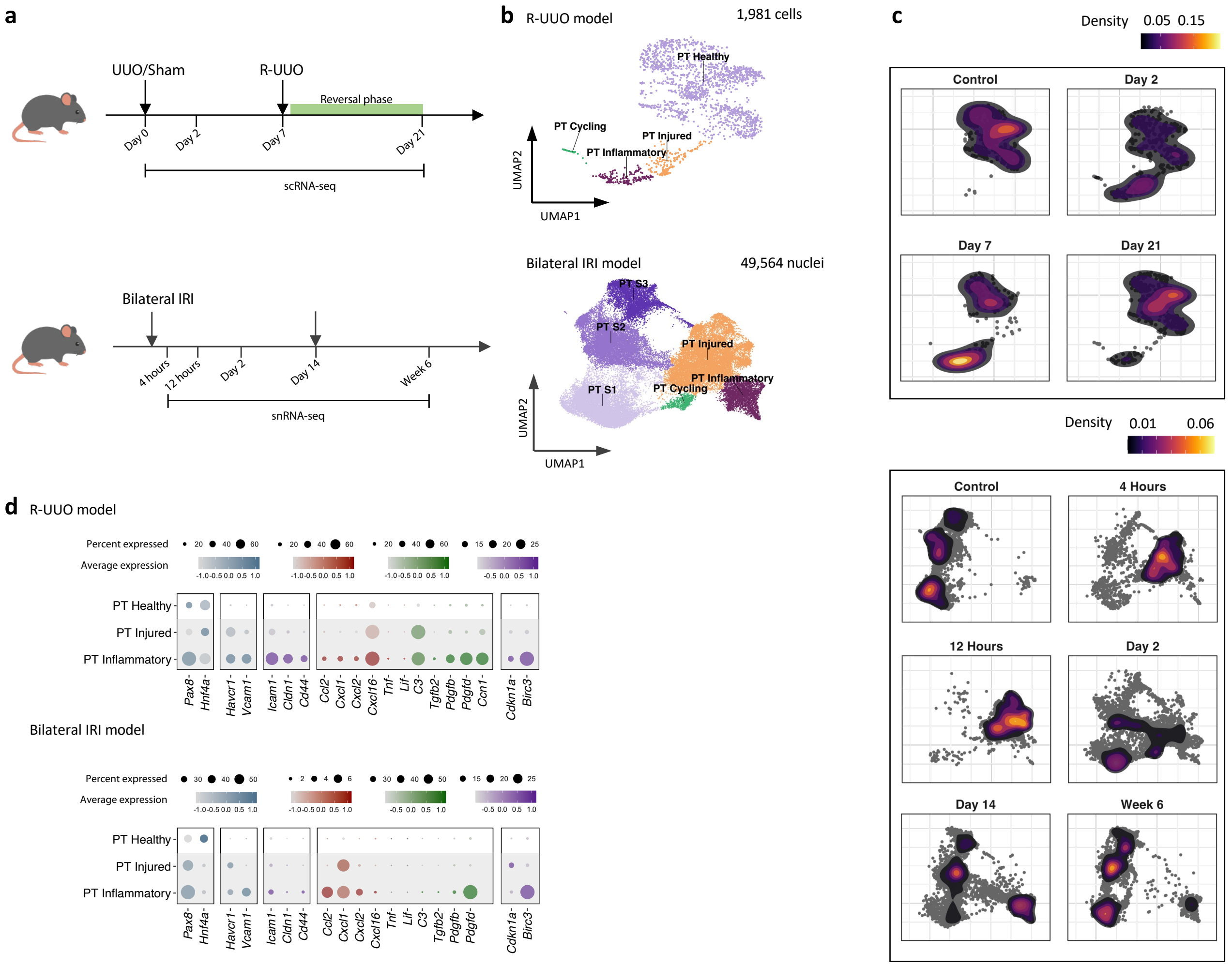

Fig. S10. Inflammatory proximal tubule cells persist after injury in mouse models of AKI.

a) Experimental summary of the murine single nucleus/cell RNA-seq datasets employed for human-murine comparison (9, 14).

b) UMAP embeddings and cell annotations of re-clustered mouse proximal tubule (PT) cells from the reversible unilateral ureteric obstruction (R-UUO) model (left) and ischaemia-reperfusion injury (IRI) model (right).

c) Density graph showing the changes in proportion of PT cell types at different timepoints following injury. Top row: R-UUO model. Bottom row: IRI model. Healthy proximal tubule cells are most predominant in control kidneys, whilst injury signatures arise early after injury. Pro-inflammatory PTs accumulate at later timepoints and persist after resolution of injury.

d) Expression patterns of human proximal tubule, injury and inflammatory markers projected onto corresponding PT cells in mice. Dot plot showing average marker expression values (log scale) and proportions of cells expressing the transcript. A conserved inflammatory, pro-fibrotic signature is conserved in both datasets. *Vcam1* is specific to inflammatory PT cells in mice, but is broadly expressed by both injured and inflammatory PT cells in humans.

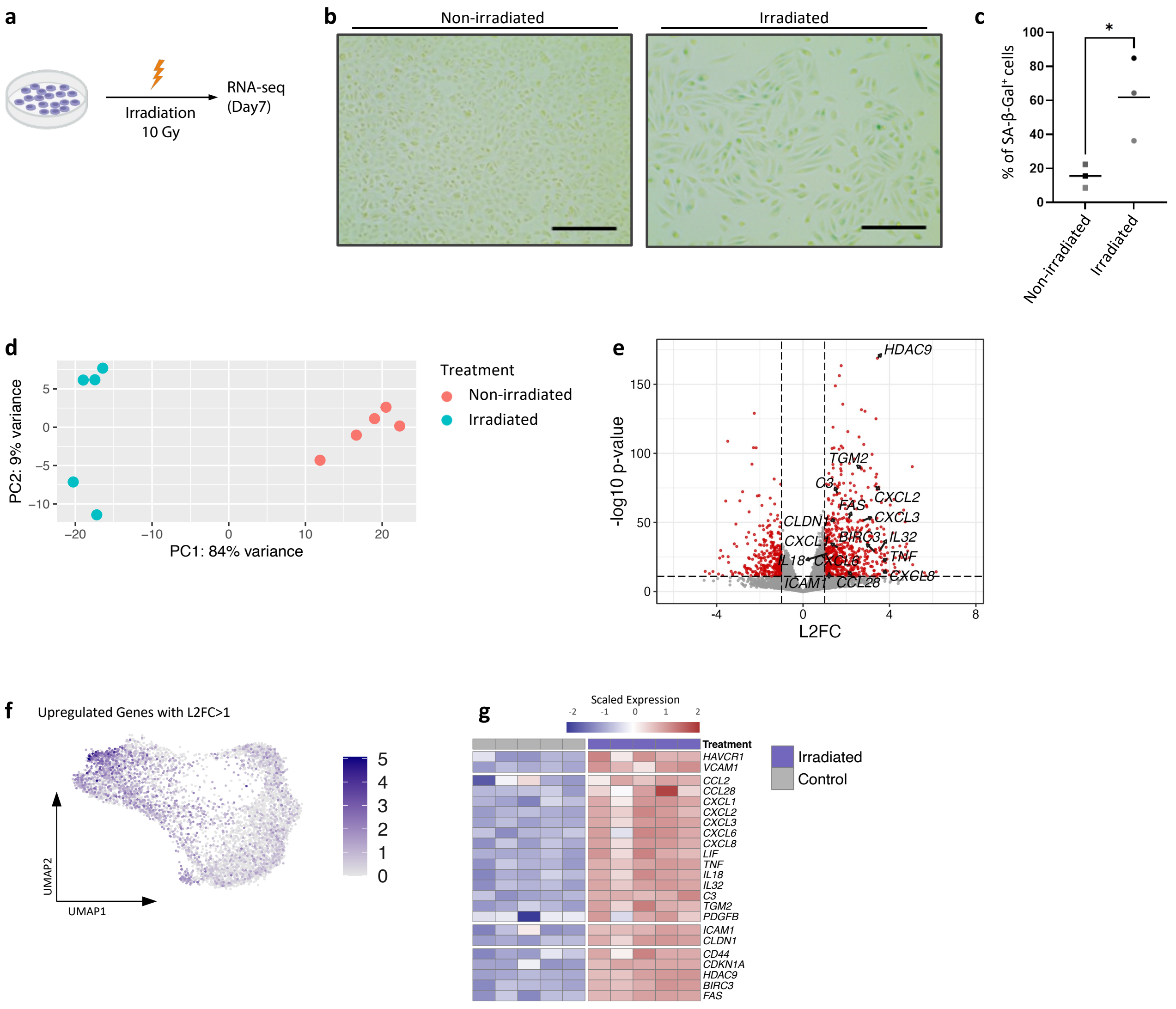

Fig. S11. Irradiation of RPTECs replicates transcriptome of inflammatory PT cells.

a) Schemata of experimental plan.

b) Representative images of Senescence-associated-β-galactosidase (SA-β-gal) staining seven days post irradiation. Scale bars, 200 µm.

c) Quantification of SA-β-Gal positive cells seven days post irradiation. Data are from 3 independent experiments and are presented as means, with comparisons assessed by students *t*-test. *$p < 0.05$

d) Principal component analysis (PCA) showing separation of irradiated and non-irradiated samples in the first principal component with 84% variance.

e) Volcano plot for the comparison between irradiated and non-irradiated samples with previously identified genes highlighted.

f) Radiation-induced senescence in human primary renal proximal tubule epithelial cells (RPTECs) promotes a gene signature comparable to that of inflammatory PT cells in vivo (**Fig. 2c**). The plots shows the gene signature score of upregulated genes (L2FC > 1, adj. $p < 0.05$) in irradiation-induced senescent RPTECs mapped onto the UMAP of human PT cells (**Fig. 2a**).

g) Heatmap of scaled gene expression in control and irradiated RPTECs, showing the gene expression changes of markers of inflammatory PT cells (**Fig. 2c**).

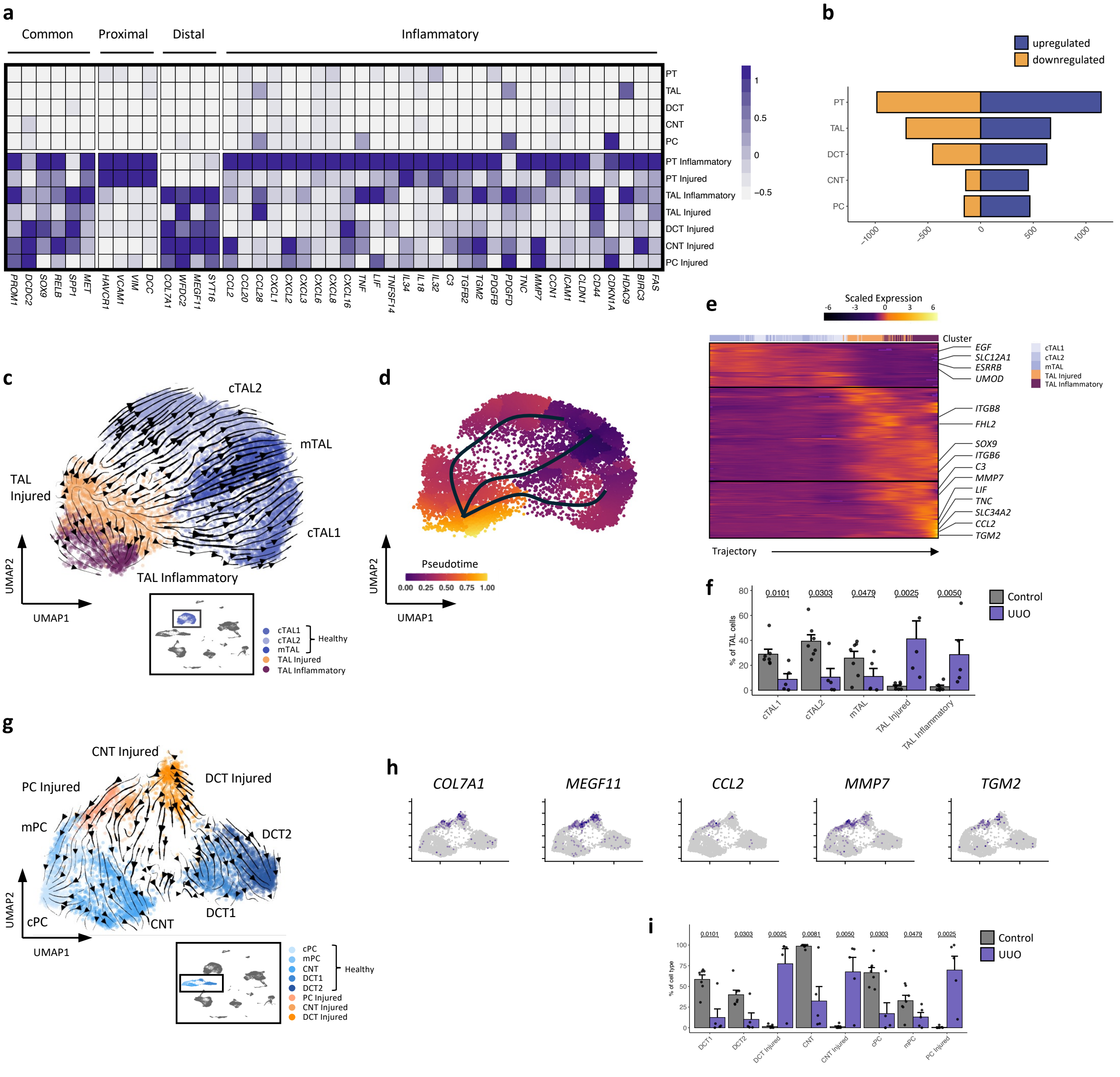

Fig. S12. Injury trajectories in non-PT nephron segments.

a) Injury and inflammatory signature (**Fig. 2c**) in different nephron segments. Tile colour indicates the average expression level in the cluster.

b) Number of differentially expressed genes (L2FC > 0.25, adj. $p < 0.05$) by nephron segment.

c) Thick ascending limb of Loop of Henle (TAL) subset of the UMAP (**Fig. 1b**) with cells coloured by subcluster annotations and projected RNA velocities derived from the most variable genes.

d) UMAP (as in **Fig. S12c**) with cells ordered in pseudotime on a trajectory from healthy TAL subclusters (cortical TAL (cTAL) 1-2, medullary TAL (mTAL)) to the inflammatory cell state.

e) Heatmap of smoothed gene expression dynamics along the inferred pseudotime trajectory (**Fig. S12d**).

f) Barplot showing the percentage of TAL subclusters in control (n = 7) and obstructed kidneys (n = 5) as a proportion of all TAL cells in the sample. Data are means ± SEM. Wilcoxon rank-sum test.

g) Distal convoluted tubule (DCT), connecting tubule (CNT) and principal cell (PC) subsets of the UMAP (**Fig. 1b**) with cells coloured by subcluster annotations and projected RNA velocities derived from the most variable genes.

h) UMAP (**Fig. S12g**) plots showing the gene expression of injury and inflammatory markers in DCT, CNT and PC clusters. Colours are scaled by the log-scale expression.

i) Barplot showing the percentage of DCT, CNT and PC subclusters in control (n = 7) and obstructed kidneys (n = 5) as a proportion of the total number of that respective cell type. Data are means ± SEM. Wilcoxon rank-sum test.

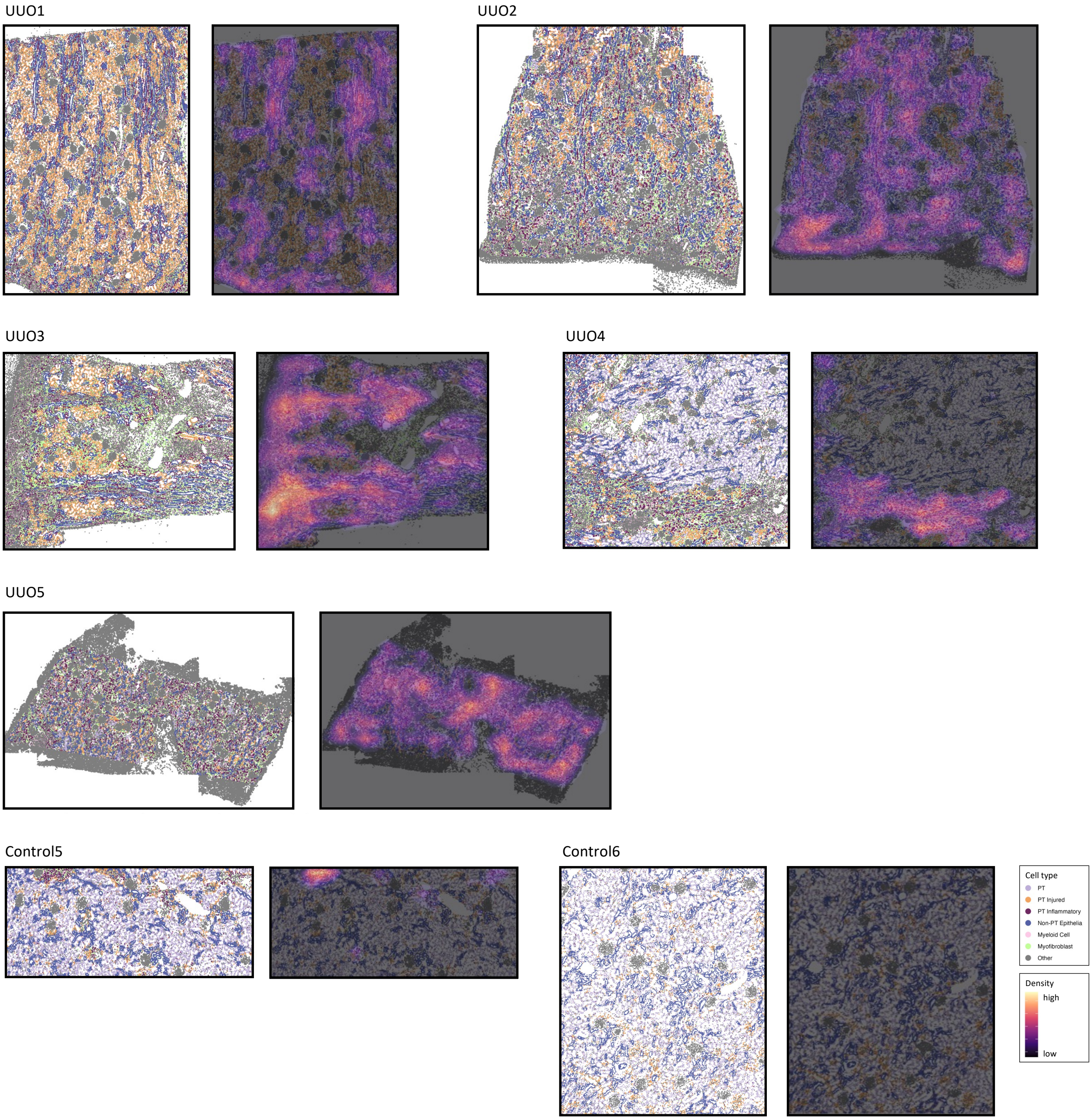

Fig. S13. Localisation of inflammatory in UUO samples.
Plots show 2D spatial plots of all CosMx (6,000-plex) UUO and control samples. Dots represent individual cells coloured according to cell types (left). Density map overlay indicating the abundance of the inflammatory PT cells (right).

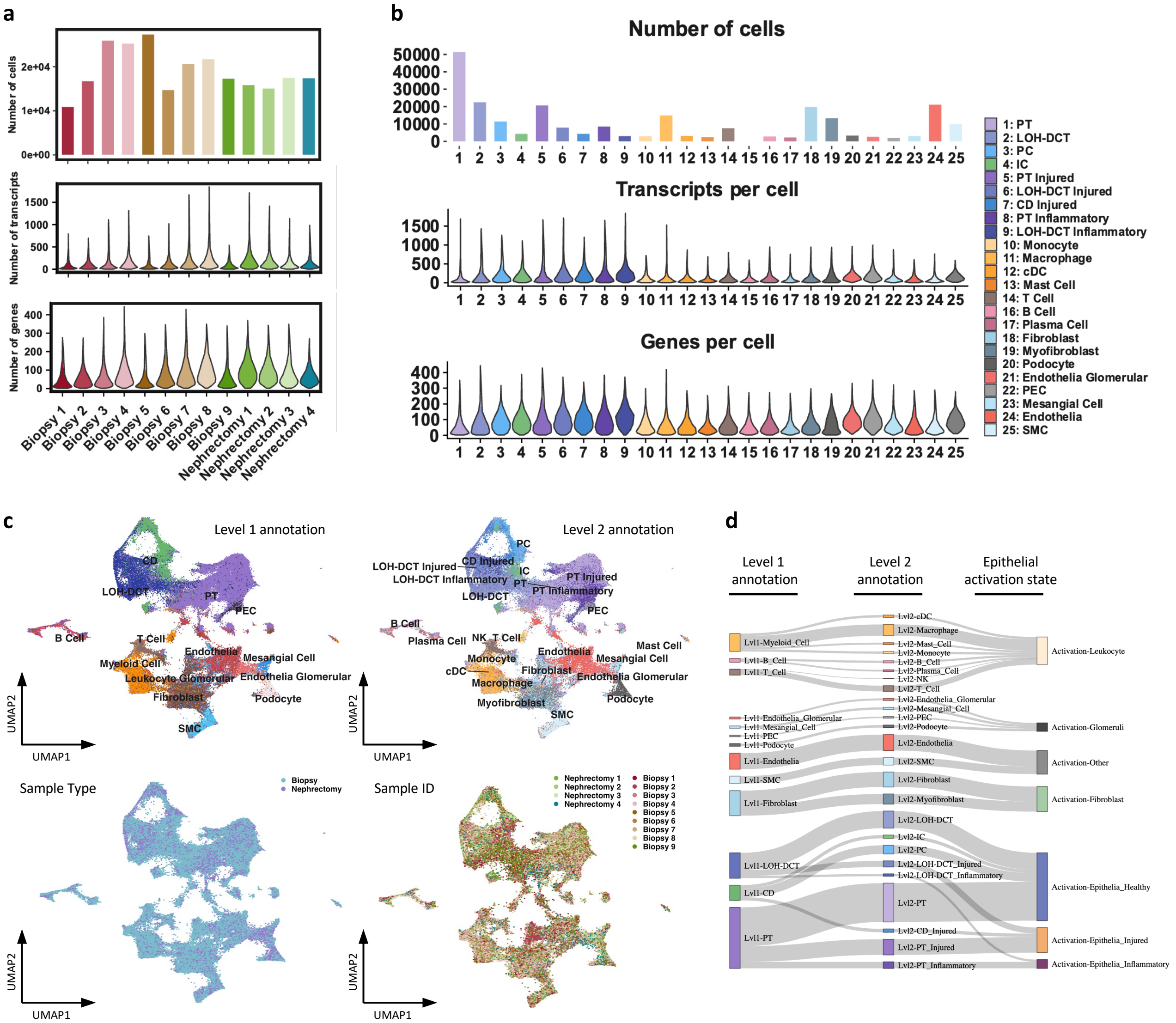

Fig. S14. Sample and cell type quality metrics in the CosMx (1,000-plex) data set.

a) Bar and violin plots showing the number of cells and the number transcripts or genes detected per biopsy or nephrectomy sample.

b) Bar and violin plots showing the number of cells and the number transcripts or genes detected grouped by cell type. PT, proximal tubule; LoH-DCT, loop of Henle-distal convoluted tubule; PC, principal cell; IC, intercalated cell; CD, collecting duct; cDC, classical dendritic cell; PEC, parietal epithelial cell; SMC vascular smooth muscle cell/pericyte.

c) UMAP (**Fig. 4b**) coloured by level1 and level2 cell type annotations (upper) and sample type or sample ID (lower).

d) Sankey diagram illustrating the relationship between level1, level2 and epithelial activation state cell annotations shown.

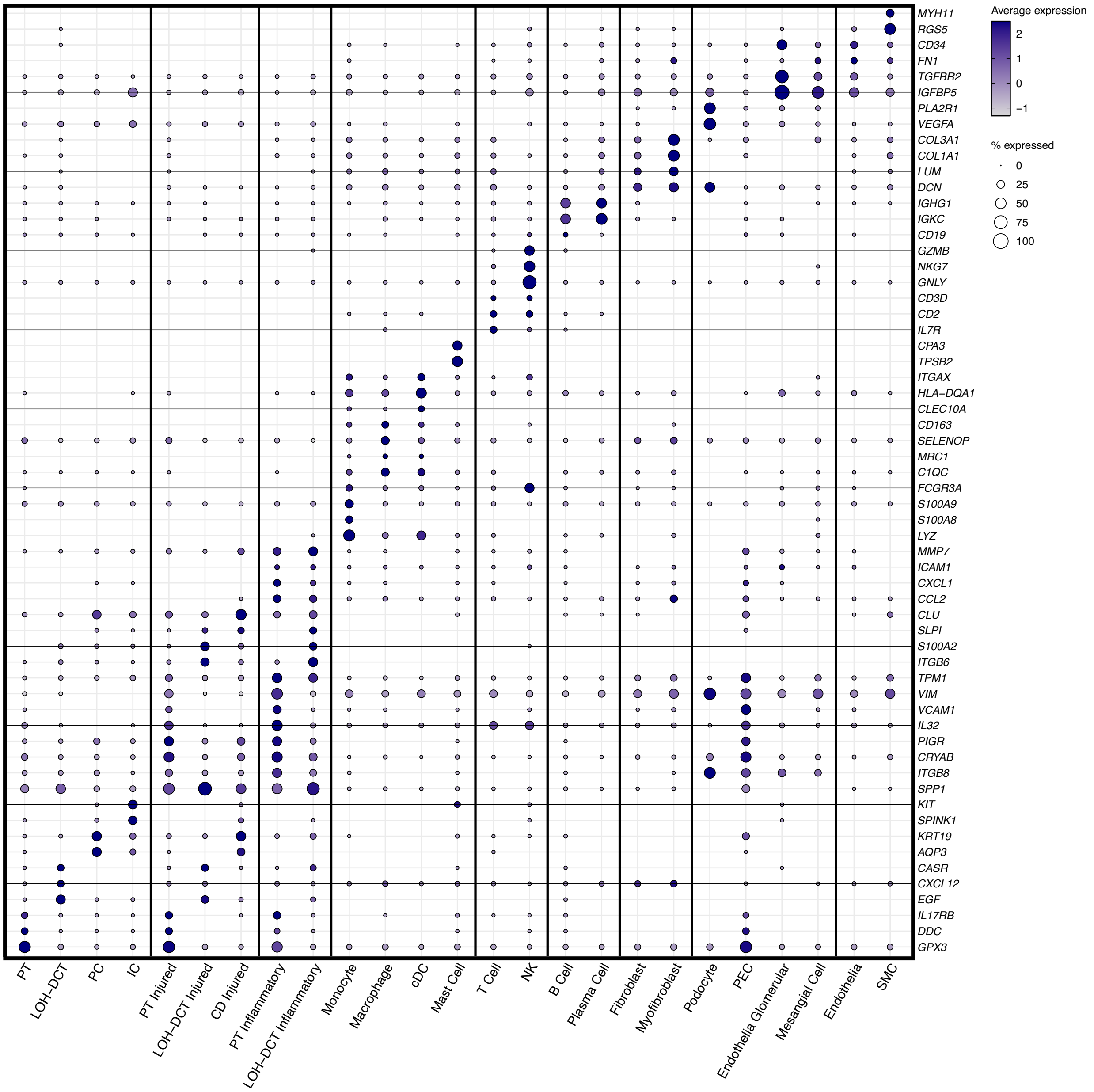

Fig. S15. CosMx (1,000-plex) cell type marker expression.

Dot plot showing the expression of marker genes used to identify cell types. Dot colours show the averaged gene expression values (log scale) and size indicates proportion of cells expressing the gene.

PT, proximal tubule; LoH-DCT, loop of Henle-distal convoluted tubule; PC, principal cell; IC, intercalated cell; CD, collecting duct; cDC, classical dendritic cell; PEC, parietal epithelial cell; SMC vascular smooth muscle cell/pericyte.

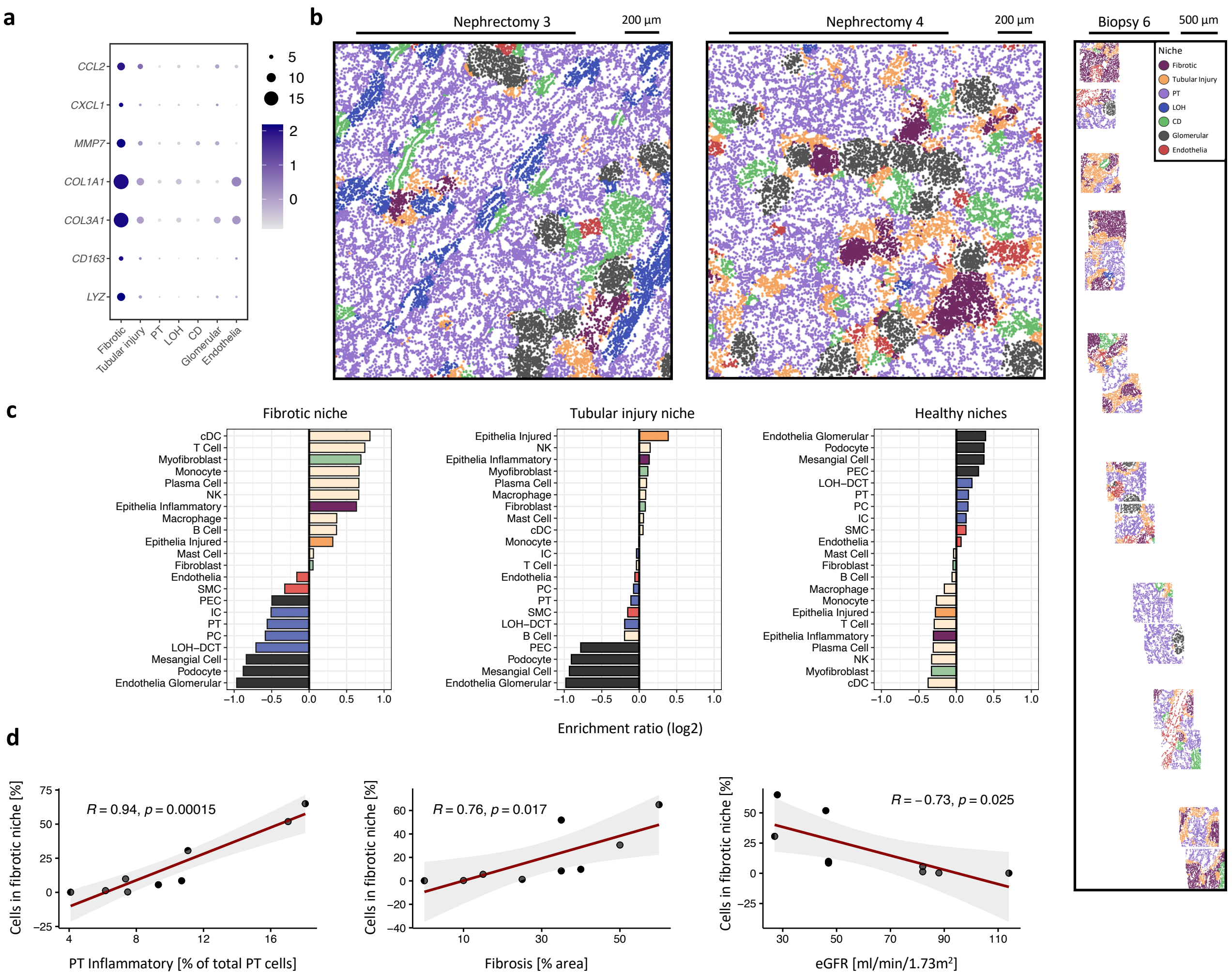

Fig. S16. Characterisation of the fibrotic niche in CKD samples (CosMx 1,000-plex panel).

a) Expression levels of inflammatory epithelia, myofibroblast and myeloid cell markers in spatial niches. The dot plot shows the average marker expression values (log scale) and proportions of cells expressing the transcript.

b) Projection of niches into 2D space for representative nephrectomy and biopsy samples. Cell segmentation boundaries and coloured according their associated niche.

c) Cell type enrichment in niches. Barplot show the log2-fold enrichment ratio of observed cell type frequencies within the niche over a random tissue distribution. Healthy niches represent PT, LOH, CD, Glomerular and Endothelia niches.

d) Correlation of the proportion of cells in the fibrotic niche with proportion of inflammatory PT cells, total fibrotic area and eGFR from n = 9 biopsies from patients with CKD. The graphs show linear regression slopes with Pearson correlation co-efficients.

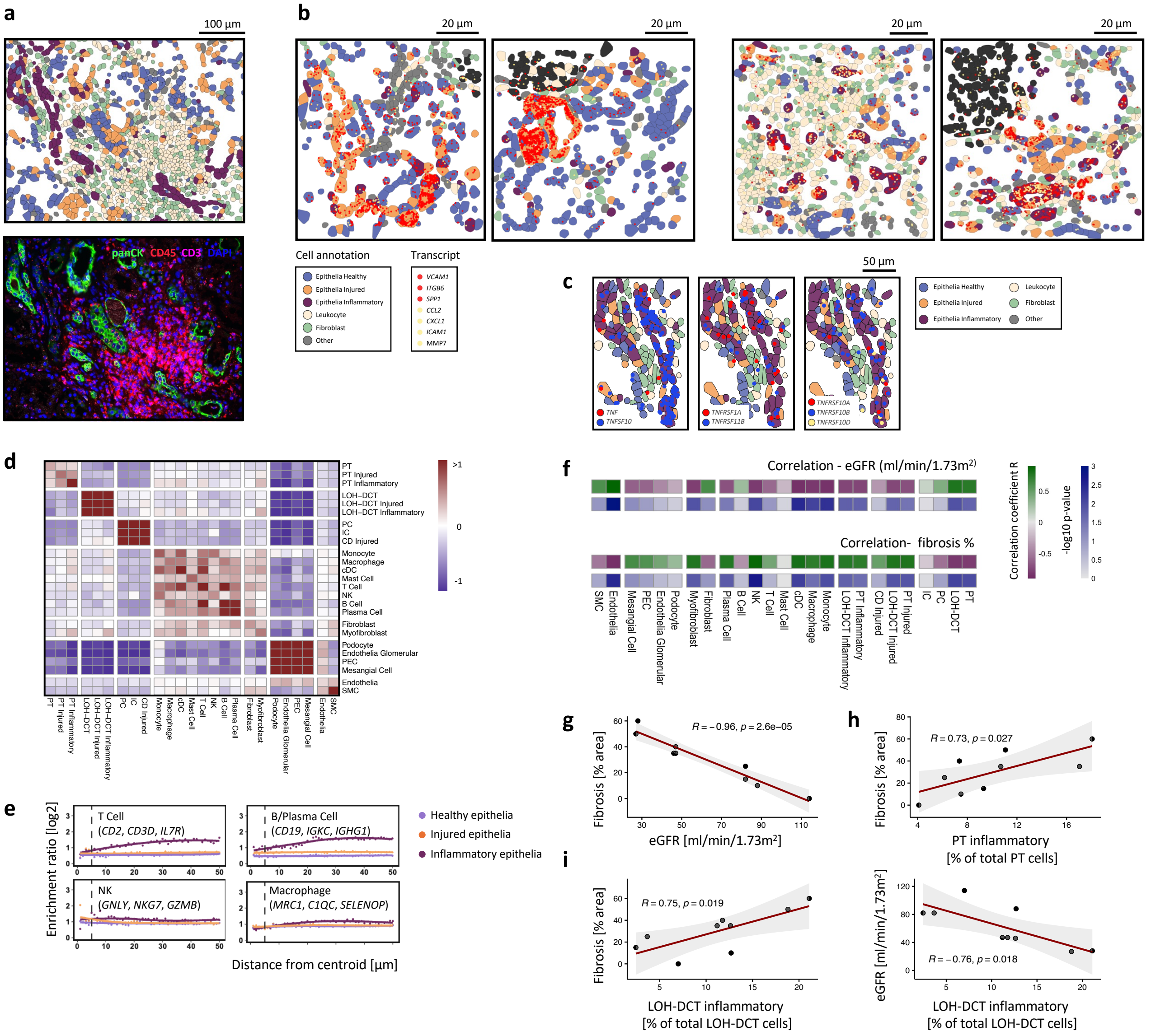

Fig. S17. CosMx extended images and correlation with clinical parameters in CKD samples (CosMx 1,000-plex panel).

a) Association of RNA-based cell type annotation and the respective multiplex immunofluorescence image. Top: Detected cells in x-y coordinates coloured by broad cell state annotation. Bottom: The same frame with multiplexed immunostaining. panCK, pancytokeratin.

b) Extended representative images of injured and inflammatory tubules and their cellular neighbourhoods in different samples. Left panel: Injured tubules (orange) expressing injury markers, but no inflammatory transcripts. Right panel: Inflamed tubules (purple) located in the fibrotic niche express combined injury and inflammatory markers. Images show the cell segmentation boundaries in their x-y coordinates coloured by epithelial activation state and broad cell types. Small dots show the location of selected injury (red) or inflammatory-related (yellow) transcripts.

c) High-resolution image of spatial expression patterns of TNF family ligands and receptors signalling between PT cells. The images show the spatial location of detected ligands (left two) and receptors (right). Cell boundaries are coloured by cell lineage and epithelial activation state.

d) Cell neighbourhoods. Heatmap of mean log2-fold enrichment (mean of all samples) showing the probability of observing each cell type within 25 µm radius of the reference cell type. An enrichment of zero indicates a ratio equal to that expected in a random distribution of cells in the tissue. Values greater than zero indicate enrichment in the neighbourhood and value less than zero indicate relative depletion. cDC, classical dendritic cell; NK, natural killer cell.

e) Plots show the enrichment ratio (mean of the indicated transcripts) in 1 µm intervals relative to centroids of a given cell type.

f) Correlation of cell type proportions with eGFR and fibrosis in biopsy samples. Epithelial (proximal tubule, PT; LOH-DCT, loop of Henle-distal convoluted tubule; principal cell, PC; intercalated cell, IC; collecting duct, CD) percentages are calculated in relation to the abundance of the given nephron segment to normalise for differences due to biopsy location. All other other cell types are considered in relation to total cells cDC, classical dendritic cell; NK, natural killer cell; PEC, parietal epithelial cell; SMC, smooth muscle cell. The heatmap shows correlation coefficients and *p*-values (-log10 scale) associated with a linear regression slope with Pearson correlation co-efficient.

g-h) Correlation between proportion of biopsy occupied by fibrosis and **g)** eGFR **h)** percentage of PT cells that have an inflammatory phenotype in renal biopsies from patients with CKD (n = 9). Graphs show linear regression slopes with Pearson correlation co-efficients.

i) Correlation between inflammatory cell states in LOH-DCT nephron segments with fibrosis area and eGFR in renal biopsies from patients with CKD (n = 9). The graphs show linear regression slopes with Pearson correlation co-efficients.

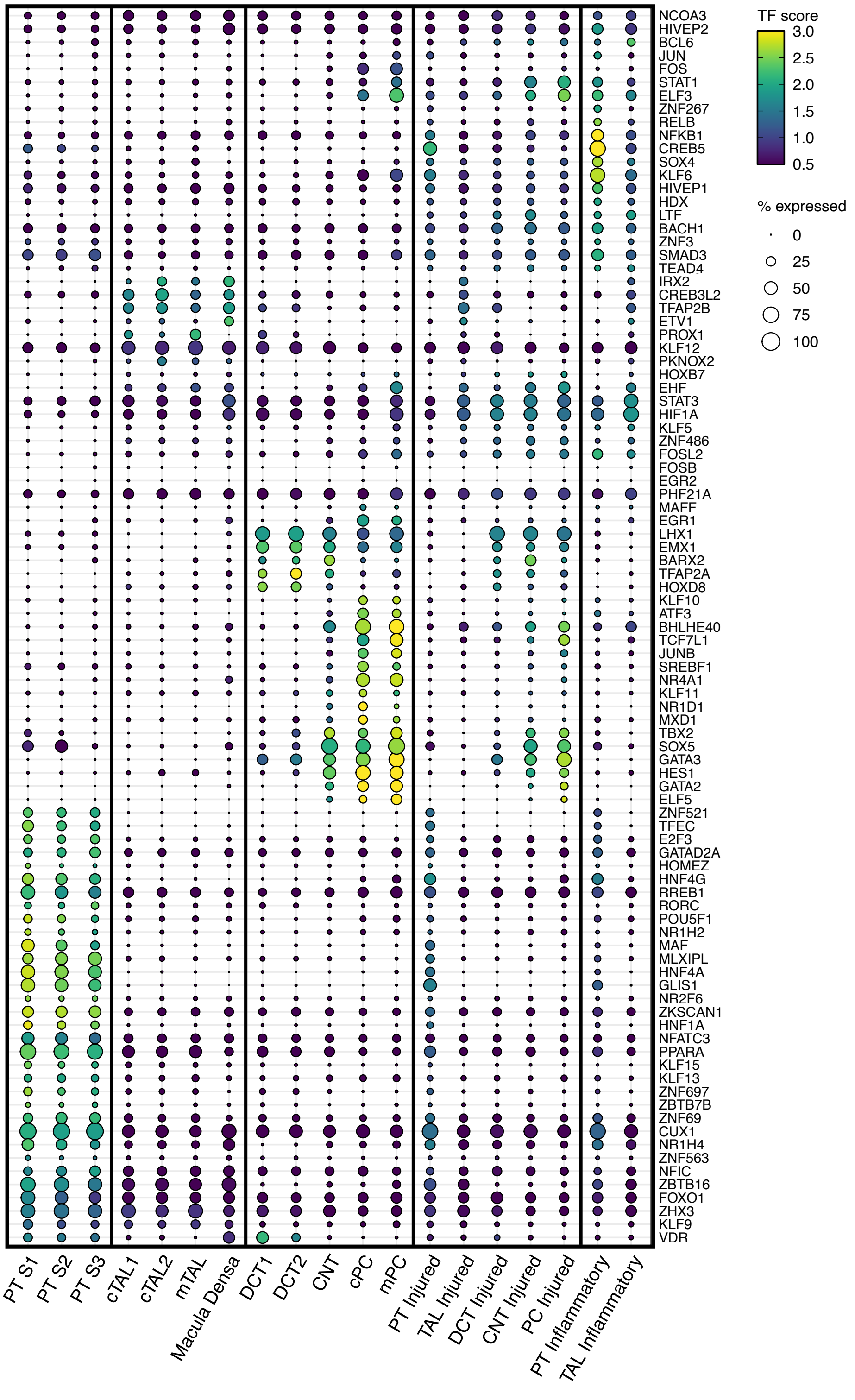

Fig. S18. Transcription factor activity in injured and inflammatory nephron segments.

Dot plots of TF activity (mean of target gene expression score and accessibility score of regulatory elements linked to each TF) and expression of TF gene expression in different nephron segments and cell states for TFs with > 50 predicted target genes. The dot colour shows the TF score and the dot size is scaled by the proportions of cells expressing the TF gene.

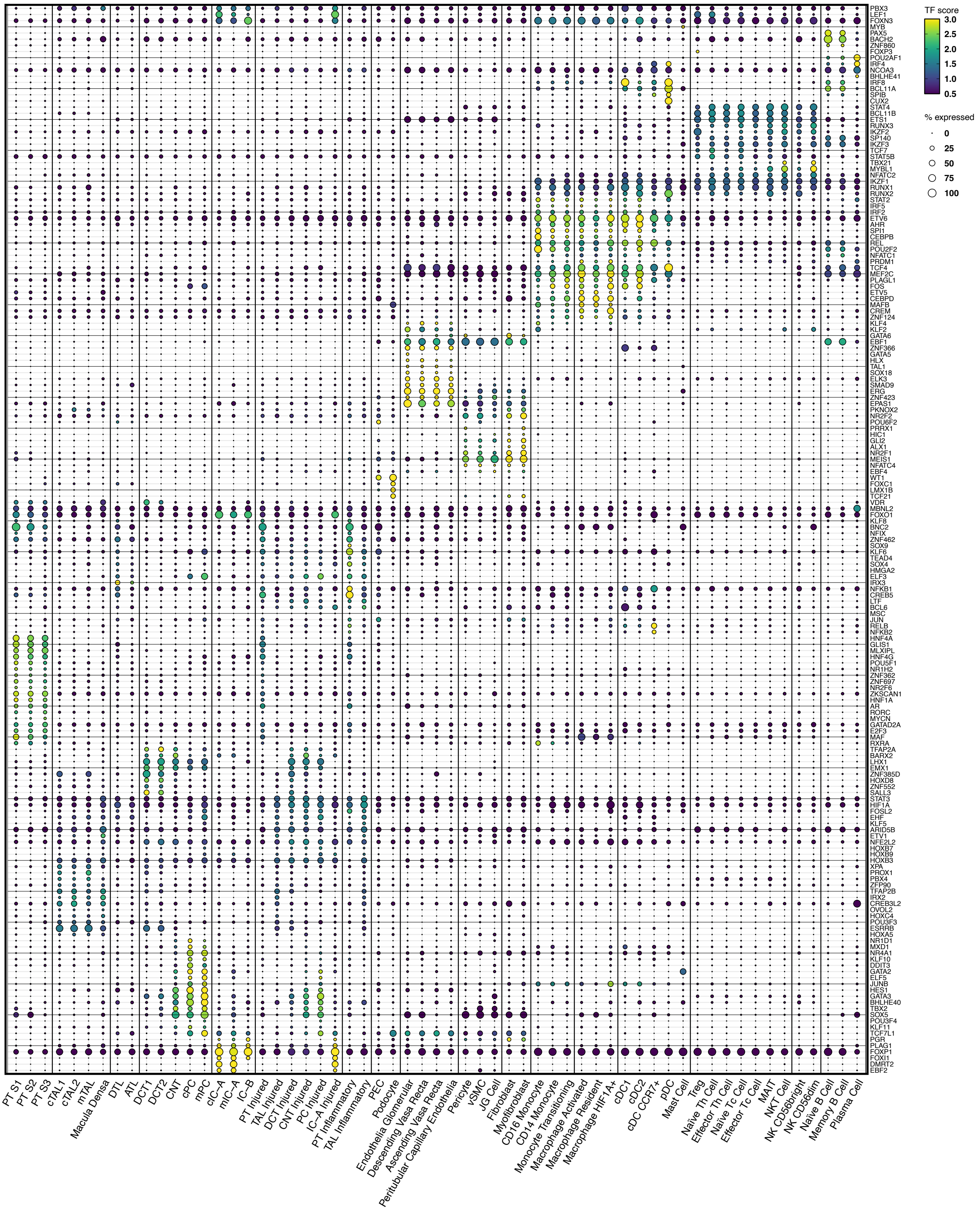

Fig. S19. Transcription factor activities in all kidney cell types.

Dot plots of transcription factor activity and expression in all cell types for TFs with > 15 target genes. The dot colour shows TF score (mean of target gene expression score and accessibility score of regulatory elements linked to each TF). The dot size is scaled by the proportions of cells expressing the TF gene.

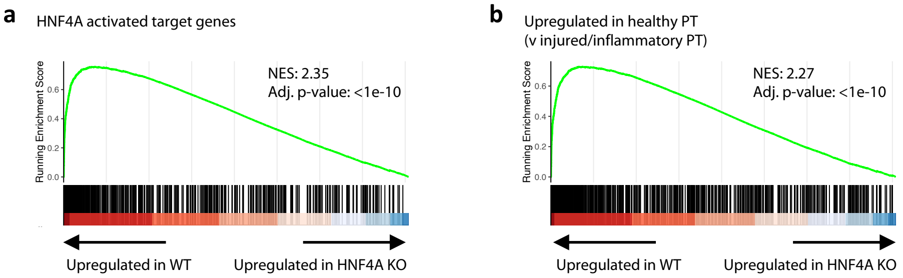

Fig. S20. Agreement with HNF4A knockout signatures in organoids.

a) Gene Set Enrichment Analysis (GSEA) plot showing that our predicted HNF4A target genes are enriched amongst genes over-expressed in WT versus HNF4A knockout human kidney organoids (30).

b) GSEA plot showing the enrichment of genes upregulated (LFC > 0.1, adj. $p < 0.05$) in healthy PT cells compared to injured and inflammatory PT cells (based on the snRNA-seq dataset presented here).

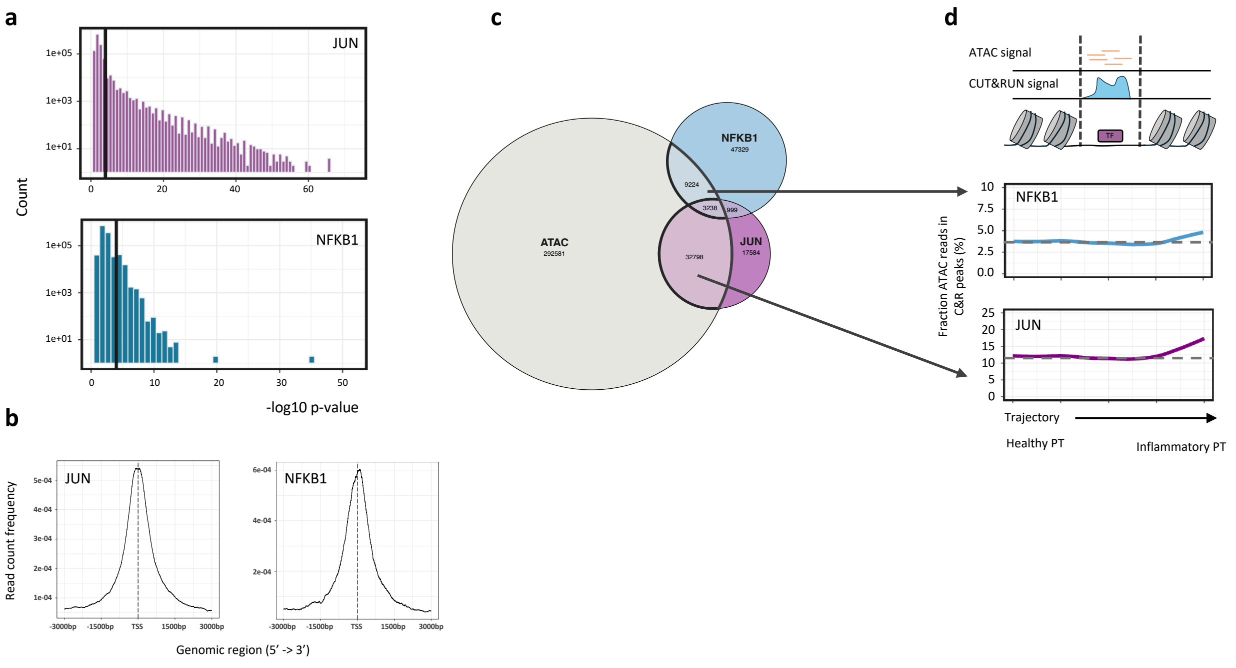

Fig. S21. Integration of snATAC-seq and CUT&RUN data.

a) Histogram of -log10 *p*-values associated with peaks called by MACS2 in CUT&RUN datasets. Vertical lines indicate the cut-off chosen for JUN or NFKB1 experiments.

b) Average profile of CUT&RUN peaks around transcription start site (TSS) regions indicating greatest binding near the TSS.

c) Venn diagram showing overlap of JUN and NFKB1 CUT&RUN with ATAC peaks (min. 10% overlap on either side).

d) Integration of ATAC and CUT&RUN data. Top: reads from snATAC-seq data are used to assess chromatin state at JUN and NFKB1 binding sites. Bottom: Fraction of ATAC reads overlapping with JUN or NFKB1 peaks along the pseudotime trajectory from healthy to inflammatory PT cells (**Fig. 2f**).

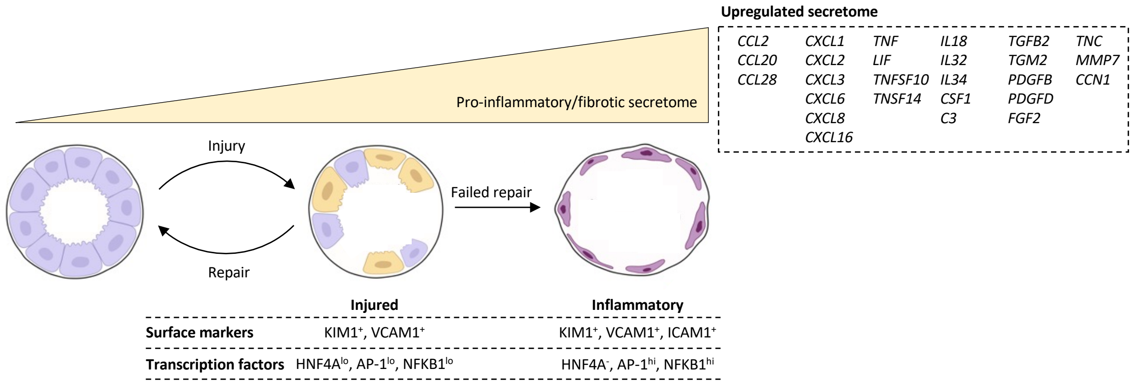

Fig. S22. Graphic summary of PT cell states.
Graphic summarising key findings. Created in BioRender. Reck, M. (2025) https://BioRender.com/m603whw
