# Supplementary material for: Multiomic analysis of human kidney disease identifies a tractable inflammatory and pro-fibrotic tubular cell phenotype
Source: Nat Commun. 2025 May 22;16:4745. doi: 10.1038/s41467-025-59997-4 (PMC12095627; doi:10.1038/s41467-025-59997-4)
Supplement: Supplementary file 2 — Description of Additional Supplementary Files [file 41467_2025_59997_MOESM2_ESM.pdf]

Description of Additional Supplementary Files

File Name: Supplementary Data 1
Description: **Clinical characteristics of multiome samples.**
Summary of clinical characteristics of patients in which snRNA/ATAC sequencing was performed on the non-tumorous portion of their tumour nephrectomy specimens. Comparisons of clinical and histological characteristics between the unobstructed and obstructed groups were performed by students t-test or Chi-test for continuous or categorical data respectively.

File Name: Supplementary Data 2
Description: **Summary of the multiome experiment.**
Summary of snRNA/ATAC-seq experiment and samples. The left panel shows detailed clinical characteristics of samples used. The middle panel shows the pooling layout with samples pooled in different libraries, with the number (percentage) of post-QC nuclei recovery shown. The right panel shows key assay performance metrics. eGFR, estimated glomerular filtration rate; UMI, unique molecular identifier.

File Name: Supplementary Data 3
Description: **Summary of multiome cluster annotations.**
Cell annotations at different resolutions for all identified clusters and respective assay performance metrics and the number of recovered nuclei. The left panel shows the relationship between different annotation levels. The middle panel shows assay performance for each cell type. The right panel shows the number (percentage of total) of recovered nuclei in each sample.

File Name: Supplementary Data 4
Description: **Multiome marker genes.**
List of the top 200 markers of each cluster sorted by log2-fold change. P-values were calculated for genes expressed by at least 3% of cells in the cluster against all other cells using a Wilcoxon rank sum test and p-values were corrected using the Benjamini–Hochberg method. L2FC, log2-fold change; adj. p-value, adjusted p-value.

File Name: Supplementary Data 5
Description: **Genes differentially expressed in healthy, injured and inflammatory tubular epithelial cells.**
Lists of differentially expressed genes between healthy, injured or inflammatory counterparts for different nephron segments. Differentially expressed genes are reported for PT (I. and II.), TAL (III. and IV.) and DCT, CNT and PC (V.-VII.) segments. P-values were calculated for genes expressed by at least 3% of cells in the cluster against all other cells using a Wilcoxon rank sum test and p-values were corrected using the Benjamini–Hochberg method. L2FC, log2-fold change; adj. p-value, adjusted p-value.

File Name: Supplementary Data 6
Description: **PT trajectory gene expression dynamics.**
Expression level of genes in the PT trajectory from healthy to inflammatory cell states. The early module corresponds to genes in healthy PT cells, the late module is upregulated in inflammatory PT cells. Numeric values indicate the proportions of cells in the cluster expressing the gene.

File Name: Supplementary Data 7
Description: **TAL trajectory gene expression dynamics.**
Expression level of genes in the TAL trajectory from healthy to inflammatory cell states. The early module corresponds to genes in healthy TAL cells, the late module is upregulated in inflammatory TAL cells. Numeric values indicate the proportions of cells in the cluster expressing the gene.

File Name: Supplementary Data 8
Description: **Shared and distinct reponses in nephron segments.**
Expression level of injury and inflammatory response genes upregulated by PT (I.), non-PT (II.) and shared (III.). Numeric values indicate the proportions of cells in the cluster expressing the gene.

File Name: Supplementary Data 9
Description: **CosMx (6,000-plex dataset) experiment summary.**
Summary of the CosMx (6,000-plex) samples and experimental setup. The left panel shows clinical characteristics of samples used. The middle panel shows the slide ID the sample was imaged on and the number of FOVs imaged. The right panel shows key assay performance metrics. eGFR, estimated glomerular filtration rate; FOV, field of view.

File Name: Supplementary Data 10
Description: **Summary of CosMx (6,000-plex dataset) cluster annotations.**
Cell annotations at different resolutions for all identified clusters and respective assay performance metrics and the number of detected cells. The left panel shows the relationship between different annotation levels. The middle panel shows assay performance for each cell type. The right panel shows the number of detected cells (percentage of total) in each sample.

File Name: Supplementary Data 11
Description: **CosMx marker genes (6,000-plex dataset).**
List of top cluster markers with an average L2FC>0.1 in the CosMx dataset. P-values were calculated for genes expressed by at least 3% of cells in the cluster against all other cells using a Wilcoxon rank sum test and p-values were corrected using the Benjamini–Hochberg method. L2FC, log2-fold change; adj. p-value, adjusted p-value.

File Name: Supplementary Data 12
Description: **CosMx (6,000-plex dataset) cell neighbourhoods.**
Numeric values showing log2-fold enrichment of cell types encountered within a 25µm radius of a given cell type. Enrichments are calculated per sample and reported as mean of all samples. An enrichment of zero indicates a ratio equal to that expected in a random

distribution of cells in the tissue. Values greater than zero indicate enrichment in the neighbourhood and value less than zero indicate relative depletion.

File Name: Supplementary Data 13
Description: **CosMx experiment summary (1,000-plex dataset).**
Summary of the CosMx samples and experimental setup. The left panel shows clinical characteristics of samples used. The middle panel shows the slide ID the sample was imaged on and the number of FOVs imaged. The right panel shows key assay performance metrics. eGFR, estimated glomerular filtration rate; FOV, field of view.

File Name: Supplementary Data 14
Description: **Summary of CosMx cluster annotations (1,000-plex dataset).**
Cell annotations at different resolutions for all identified clusters and respective assay performance metrics and the number of detected cells. The left panel shows the relationship between different annotation levels. The middle panel shows assay performance for each cell type. The right panel shows the number of detected cells (percentage of total) in each sample.

File Name: Supplementary Data 15
Description: **CosMx marker genes (1,000-plex dataset).**
List of the top 20 cluster markers (sorted by L2FC) with an average L2FC>0.1 in the CosMx dataset. P-values were calculated for genes expressed by at least 3% of cells in the cluster against all other cells using a Wilcoxon rank sum test and p-values were corrected using the Benjamini–Hochberg method. L2FC, log2-fold change; adj. p-value, adjusted p-value.

File Name: Supplementary Data 16
Description: **Epithelia differentially expressed genes in the CosMx 1,000-plex dataset.**
Lists of differentially expressed genes with an average L2FC>0.1 between healthy cells and injured or inflammatory counterparts for different nephron segments in the CosMx dataset. Differentially expressed genes are reported for PT (I. and II.), LOH and DCT (III. and IV.) and CD (V.) segments. P-values were calculated for genes expressed by at least 3% of cells in the cluster against all other cells using a Wilcoxon rank sum test and p-values were corrected using the Benjamini–Hochberg method. L2FC, log2-fold change; adj. p-value, adjusted p-value.

File Name: Supplementary Data 17
Description: **CosMx (1,000-plex dataset) cell neighbourhoods.**
Numeric values showing log2-fold enrichment of cell types encountered within a 25µm radius of a given cell type. Enrichments are calculated per sample and reported as mean of all samples. An enrichment of zero indicates a ratio equal to that expected in a random distribution of cells in the tissue. Values greater than zero indicate enrichment in the neighbourhood and value less than zero indicate relative depletion.

File Name: Supplementary Data 18
Description: **Correlation between cell type abundance and clinical parameters (1,000-plex dataset).**
Correlation of eGFR or percentage of fibrosis area with abundance of cell types. Epithelial cell proportions are calculated in relation to the abundance of the given nephron segment to normalise for differences in kidney regions captured. Other cell proportions are calculated in relation to all cells. The table shows Pearson correlation coefficients and associated p-values. eGFR, estimated glomerular filtration rate.

File Name: Supplementary Data 19
Description: **Ligand and Receptor expression patterns.**
Expression level of reported ligand and receptor pairs in the datasets. Numeric values indicate the percentage of cells in the cluster expressing the gene.

File Name: Supplementary Data 20
Description: **Multiome marker peaks.**
List of the top 200 cluster-specific peaks sorted by log2-fold change in peak accessibility. P-values were calculated for all peaks against the accessibility in other cells using the Wilcoxon rank sum test and p-values were corrected using the Benjamini–Hochberg method. Peaks were annotated to the closest gene by genomic coordinate and peaks without gene annotation were removed. L2FC, log2-fold change; adj. p-value, adjusted p-value.

File Name: Supplementary Data 21
Description: **Chromvar motif enrichment.**
Variations of chromatin accessibility at TF binding sites. Motif scores were calculated using the Chromvar wrapper in Signac using the homo sapiens JASPAR2020 motif collection. Differential motif activity in the cell type of interest compared with all other cell types was derived from motif scores was assessed using the Wilcoxon rank sum test and p-values were corrected using the Benjamini–Hochberg method. adj. p-value, adjusted p-value.

File Name: Supplementary Data 22
Description: **SCENIC+ regulons.**
Gene regulatory network inferred using SCENIC+. The left table contains all target region-to-gene links with associated TF binding sites. Rho values indicate the correlation of gene expression with linked regions. The middle table shows the top 30 TFs with an average L2FC>0.1 in the cell of interest compared with all other cell types (determined by scoring the accessibility of target regions of each TF). The right table shows the top 30 TFs with an average L2FC>0.1 in their respective gene expression in the cell type of interest compared with all other cell types. P-values were calculated for differences in scores using the Wilcoxon rank sum test and p-values were corrected using the Benjamini–Hochberg method. TF, transcription factor; L2FC, log2-fold change; adj. p-value, adjusted p-value.

File Name: Supplementary Data 23
Description: **Antibody table.**
Table of Antibody details

File Name: Supplementary Data 24
Description: **TaqMan probe table.**
Table of TaqMan probes used.
