# Supplementary material for: Multiomic analysis of human kidney disease identifies a tractable inflammatory and pro-fibrotic tubular cell phenotype
Source: Nat Commun. 2025 May 22;16:4745. doi: 10.1038/s41467-025-59997-4 (PMC12095627; doi:10.1038/s41467-025-59997-4)
Supplement: Supplementary file 4 — Reporting Summary [file 41467_2025_59997_MOESM4_ESM.pdf]

nature portfolio

Corresponding author(s): Bryan Conway (bryan.conway@ed.ac.uk)

Last updated by author(s): Apr 02, 2025

Reporting Summary

Nature Portfolio wishes to improve the reproducibility of the work that we publish. This form provides structure for consistency and transparency in reporting. For further information on Nature Portfolio policies, see our Editorial Policies and the Editorial Policy Checklist.

Statistics

For all statistical analyses, confirm that the following items are present in the figure legend, table legend, main text, or Methods section.

n/a	Confirmed	
☐	☒	The exact sample size (*n*) for each experimental group/condition, given as a discrete number and unit of measurement
☐	☒	A statement on whether measurements were taken from distinct samples or whether the same sample was measured repeatedly
☐	☒	The statistical test(s) used AND whether they are one- or two-sided<br>*Only common tests should be described solely by name; describe more complex techniques in the Methods section.*
☐	☒	A description of all covariates tested
☐	☒	A description of any assumptions or corrections, such as tests of normality and adjustment for multiple comparisons
☐	☒	A full description of the statistical parameters including central tendency (e.g. means) or other basic estimates (e.g. regression coefficient) AND variation (e.g. standard deviation) or associated estimates of uncertainty (e.g. confidence intervals)
☐	☒	For null hypothesis testing, the test statistic (e.g. *F*, *t*, *r*) with confidence intervals, effect sizes, degrees of freedom and *P* value noted<br>*Give P values as exact values whenever suitable.*
☐	☒	For Bayesian analysis, information on the choice of priors and Markov chain Monte Carlo settings
☐	☒	For hierarchical and complex designs, identification of the appropriate level for tests and full reporting of outcomes
☐	☒	Estimates of effect sizes (e.g. Cohen's *d*, Pearson's *r*), indicating how they were calculated

Our web collection on statistics for biologists contains articles on many of the points above.

Software and code

Policy information about availability of computer code

Data collection	The data collection methodology is fully described in the methods section. For data generated in this study (e.g. snRNA/ATAC-seq, CosMx spatial transcriptomics) commercial and open source software was used to process the raw sequencing or imaging data. All datasets are available as raw and processed data as indicated in the 'Data availability' section.
Data analysis	All analysis performed is described in detail in the methods section. Additionally, all code needed to re-produce figures is available on GitHub and Zenodo as indicated in the 'Code availability' section.<br>Github repository: https://github.com/mreck1/human_kidney_multiomics<br>Zenodo repository: https://zenodo.org/records/15124887

For manuscripts utilizing custom algorithms or software that are central to the research but not yet described in published literature, software must be made available to editors and reviewers. We strongly encourage code deposition in a community repository (e.g. GitHub). See the Nature Portfolio guidelines for submitting code & software for further information.

Data

Policy information about availability of data

All manuscripts must include a data availability statement. This statement should provide the following information, where applicable:

- Accession codes, unique identifiers, or web links for publicly available datasets
- A description of any restrictions on data availability
- For clinical datasets or third party data, please ensure that the statement adheres to our policy

The raw and processed data generated in this study have been deposited on NCBI Gene Expression Omninbus (GEO).
-Multiome snRNA/snATAC-seq data is available under the accession GSE254185 https://0-www-ncbi-nlm-nih-gov.brum.beds.ac.uk/geo/query/acc.cgi?acc=GSE254185
-CosMx 6,000-plex pipeline outputs and processed data is available under accession GSE282059 https://0-www-ncbi-nlm-nih-gov.brum.beds.ac.uk/geo/query/acc.cgi?acc=GSE282059
-CosMx 1,000-plex pipeline outputs and processed data is available under accession GSE253439 https://0-www-ncbi-nlm-nih-gov.brum.beds.ac.uk/geo/query/acc.cgi?acc=GSE253439
-Bulk RNA-seq data of irradiated RPTECs GSE252584 https://0-www-ncbi-nlm-nih-gov.brum.beds.ac.uk/geo/query/acc.cgi?acc=GSE252584
- CUT&RUN data can be found under accessions GSE254187 https://0-www-ncbi-nlm-nih-gov.brum.beds.ac.uk/geo/query/acc.cgi?acc=GSE254187
-Interactive visualisation of multiome snRNA-seq data is available on the CELLxGENE platform https://cellxgene.cziscience.com/e/867757c1-3b1a-49d9-a0cd-17767eb160cc.cxg
-Additional data is available on Zenodo under the accession code 15124887 https://zenodo.org/records/15124887
-Referenced data sets for mouse R-UUO is available on GEO under the accessions GSE140023 https://www.ncbi.nlm.nih.gov/geo/query/acc.cgi?acc=GSE140023
-Referenced data sets for mouseIRI is available on GEO under the accessions GSE139107 https://www.ncbi.nlm.nih.gov/geo/query/acc.cgi?acc=GSE139107
-Source data are provided with this paper

Research involving human participants, their data, or biological material

Policy information about studies with human participants or human data. See also policy information about sex, gender (identity/presentation), and sexual orientation and race, ethnicity and racism.

Reporting on sex and gender	The metadata and individual participant data for sex and self-reported sex are given in Supplementary Tables 1 and 2, respectively.
Reporting on race, ethnicity, or other socially relevant groupings	We did not collect ethnicity data. We did not exclude any ethnicities, however >98% of the population in Lothian are of white ethnicity.
Population characteristics	The metadata and individual participant data for pre- and post-op eGFR and pathological details are provided in Supplementary Tables 1 and 2, respectively.
Recruitment	Patients were recruited from a single tertiary referral urological centre by the consultant urologist. All patients were undergoing radical nephrectomy for urothelial cancer. We excluded all patients with eGFR <75ml/min/1,73m2.
Ethics oversight	Human studies were approved by the National Research Scotland Lothian Bioresource (REC 20/ES/0061), with approval for specific studies (SR1651, SR1887).

Note that full information on the approval of the study protocol must also be provided in the manuscript.

Field-specific reporting

Please select the one below that is the best fit for your research. If you are not sure, read the appropriate sections before making your selection.

☒ Life sciences ☐ Behavioural & social sciences ☐ Ecological, evolutionary & environmental sciences

For a reference copy of the document with all sections, see nature.com/documents/nr-reporting-summary-flat.pdf

Life sciences study design

All studies must disclose on these points even when the disclosure is negative.

Sample size	For the ischaemia-reperfusion study we employed n=8 animals/group, based on power to detect a 20% difference in fibrosis between the treatment groups.
Data exclusions	One animal in the ischaemia-reperfusion grouped treated with vehicle was excluded due to evidence of technical failure at the time of surgery, with no evidence of successful ischemia generation at the time of surgery or on histological or qPCR analysis.
Replication	A number of measures were analysis to confirm the validity of results (e.g. Kidney weights and PSR staining to assess for atrophy and fibrosis, respectively). There was a strong correlation (r=-0.77, p=0.001) between these measures to provide confidence in the approach. In addition, multiple inflammation/fibrosis genes were assessed.

Randomization	All mice were operated on prior to assignment to T-5224 or vehicle alone on a whole-cage basis (rotating cages for each mouse, with the operator unaware of subsequent therapy), with equal numbers of cages and mice in each group and subsequent daily dosing by gavage performed by trained animal facility staff.
Blinding	The person performing the analysis of the data was blinded to the experimental group the animals were allocated to.

Reporting for specific materials, systems and methods

We require information from authors about some types of materials, experimental systems and methods used in many studies. Here, indicate whether each material, system or method listed is relevant to your study. If you are not sure if a list item applies to your research, read the appropriate section before selecting a response.

Materials & experimental systems

n/a	Involved in the study	
☐	☒	Antibodies
☐	☒	Eukaryotic cell lines
☒	☐	Palaeontology and archaeology
☐	☒	Animals and other organisms
☒	☐	Clinical data
☒	☐	Dual use research of concern
☒	☐	Plants

Methods

n/a	Involved in the study	
☒	☐	ChIP-seq
☐	☒	Flow cytometry
☒	☐	MRI-based neuroimaging

Antibodies

Antibodies used	All antibodies used in this study are listed in Supplementary Table 23 including application, manufacturer, dilution and target species.
Validation	Below is a list of all available validation information provided by manufacturers. Additionally, for IHC/IF applications staining patterns were verified by comparison with expected results based on snRNA-seq RNA expression patterns in respective cell types.<br><br>VCAM1 EPR5047: KO Validated (manufacturer), 310 references (based on manufacturer website).<br>HAVCR1 MAB1817: Tested in WB, FC, ELISA, widely used in IHC/IF applications to stain KIM-1 in mouse kidneys, 22 references (based on manufacturer website).<br>ICAM1 HPA004877: Advanced validation (Human Protein Atlas), 6 references (based on manufacturer website).<br>PanCK (Anti-Cytokeratin, pan) C2562: Mixture of monoclonal anti-cytokeratin antibodies. It recognizes human cytokeratins 1,4,5,6,8,10,13,18 and 19. 162 references (based on manufacturer website).<br>FAP EPR20021: IHC TMA validation (manufacturer). 85 referenced (based on manufacturer website).<br>CD68 14-0688-82: Advanced verification verified by cell treatment. 43 references (based on manufacturer website).<br>IBA1 PA5-27436: Advanced verification verified by cell treatment. 59 references (based on manufacturer website).<br>JUN/c-Jun 13-2019: Tested in CUT&RUN, IHC, IP, WB. 1 references (based on manufacturer website).<br>NFKB1 13586S: Tested in CUT&RUN, ChIP, IHC, IF, WB. 142 references (based on manufacturer website).

Eukaryotic cell lines

Policy information about cell lines and Sex and Gender in Research

Cell line source(s)	RPTEC/TERT1: ATCC CRL-4031, male donor.
Authentication	No STR authentication.<br>Verification of cell morphology based on manufacturer resources. No long-term culture was used (all experiments were performed at <6 passages).
Mycoplasma contamination	Cell lines tested negative mycoplasma.
Commonly misidentified lines<br>(See ICLAC register)	None.

Animals and other research organisms

Policy information about studies involving animals; ARRIVE guidelines recommended for reporting animal research, and Sex and Gender in Research

Laboratory animals	All mice were C57BL/6J. All mice were aged 6-8 weeks.

Wild animals	This study did not involve wild animals.
Reporting on sex	The severity of the ischaemia-reperfusion injury is sex dependent, there we restricted our analysis to male mice.
Field-collected samples	This study did not involve field-collected samples.
Ethics oversight	Animal studies were approved by the University of Edinburgh Ethics Committee.

Note that full information on the approval of the study protocol must also be provided in the manuscript.

Plants

Seed stocks	This study did not involve plants.
Novel plant genotypes	This study did not involve plants.
Authentication	This study did not involve plants.

Flow Cytometry

Plots

Confirm that:

☒ The axis labels state the marker and fluorochrome used (e.g. CD4-FITC).

☒ The axis scales are clearly visible. Include numbers along axes only for bottom left plot of group (a 'group' is an analysis of identical markers).

☒ All plots are contour plots with outliers or pseudocolor plots.

☒ A numerical value for number of cells or percentage (with statistics) is provided.

Methodology

Sample preparation	Usage of flow cytometry is limited to FACS-based nuclei purification for snRNA/ATAC-seq studies. A description of methods can be found in the methods section.
Instrument	BD FACSAria Fusion (FACSAria III).
Software	Gating and sorting was performed using the BD FACSDiva (Software Version 8.0.1). No additional downstream analysis was performed.
Cell population abundance	7-AAD+ nuclei (post-size gating(comprised approximately 10% of total events. Nuclei were subsequently used for snRNA/ATAC-seq with approximately 50% of sorted nuclei being recovered as sequencing data.
Gating strategy	The gating strategy to sort nuclei is shown in Fig.S2b. Initial populations were size gated (FSC-H vs FSC-A and SSC-A vs FSC-A) and 7-AAD (DNA dye) intensity was used to identify nuclei. The 7-AAD gate was set using unstained samples.

☒ Tick this box to confirm that a figure exemplifying the gating strategy is provided in the Supplementary Information.
